# Using a systems thinking approach to explore the complex relationships between schizophrenia and premature mortality

**DOI:** 10.1177/00207640231194477

**Published:** 2023-08-29

**Authors:** Suhailah Ali, Eryn Wright, Fiona Charlson

**Affiliations:** 1 School of Public Health, The University of Queensland, Brisbane, Australia; 2 Queensland Centre for Mental Health Research, Brisbane, Australia

**Keywords:** Schizophrenia, mortality, systems thinking, social determinants, severe mental disorders, psychotic disorders

## Abstract

**Background:**

People with schizophrenia have a higher risk of mortality compared to the general population, which has not improved over time. The majority of premature deaths are due to comorbid physical diseases, driven by interrelated factors operating at the individual level, through health systems and influenced by social determinants of health. A holistic understanding of this problem and the causal pathways linking these factors together is lacking.

**Aims:**

This study aims to understand why the mortality gap between people with schizophrenia and the general population is not improving by developing a causal loop diagram (CLD), a systems thinking approach which enables empirical research and theoretical knowledge to be combined into a visual representation of causal relationships and feedback loops.

**Method:**

The CLD was constructed using published literature, including both quantitative and qualitative studies, to identify key variables and relationships, and refined through consultation with experts in the topic area.

**Results:**

A total of 21 variables and 68 connections were included in the CLD, with 23 distinct feedback loops identified. Stigma and social support had the most connections, while unemployment was involved in the greatest number of feedback loops. Most feedback mechanisms served to reinforce behavioural risk factors, inadequate healthcare and social disadvantage.

**Conclusions:**

The CLD has created a holistic and dynamic understanding of the causal pathways driving the mortality gap between people with schizophrenia and the general population, providing insights into why it has persisted over time. One of the key findings was the critical role of social determinants in shaping health outcomes, highlighting the need for person-centred models of care and multisectoral government action.

## Introduction

People with schizophrenia have a significantly reduced life expectancy compared to the general population, primarily due to comorbid physical diseases (Crump et al., 2013; Hjorthøj et al., 2017). A great deal of epidemiological research has shed light on this issue, particularly demonstrating elevated mortality risk, with 135 cohort studies spanning 1957 to 2021 included in the most recent meta-analysis on mortality in people with schizophrenia (Correll et al., 2022). However, the mortality gap, particularly in terms of deaths from natural causes, continues to persist (Ali et al., 2022; Laursen et al., 2018).

There is a general understanding of why this mortality gap exists; people with schizophrenia have an increased prevalence of behavioural risk factors such as smoking and poor diet, there are issues with healthcare provision, and they experience disparities in social and economic conditions (Liu et al., 2017). However, a holistic understanding of this problem and the causal pathways linking these factors together is lacking. There is inherent complexity involved: the factors not only operate at different levels – health status and behaviours at the individual level; factors related to health systems and services; social determinants of health – but also interact. For example, a person’s income affects their ability to eat healthy food and access primary care (Ross et al., 2015; van Zonneveld et al., 2022). These interactions are not captured in individual epidemiological studies that focus on finding a linear relationship between cause and effect.

Systems thinking is an approach aimed at understanding how parts of a system interact to form a whole and assessing dynamic complexity, that is how the behaviour of a system arises from the interactions of its parts over time (Arnold & Wade, 2015).

When we attempt to understand and address a problem that we are aware of, we often rely on our own interpretations or ‘mental models’ of what factors are influencing the problem and what we think will fix it. Rather than relying on these implicit models that contain hidden assumptions, systems thinking utilises explicit models, allowing us to test assumptions about underlying mechanisms (Cerda & Keyes, 2019; Peters, 2014). Premature mortality in people with schizophrenia has so far been primarily framed in traditional epidemiological terms, with a focus on assessing the relationship between ‘independent’ variables and outcomes of interest; by conceptualising this issue as a complex system, we can explore the interactions between the factors within this system rather than examining them in isolation. Applying a systems thinking approach allows us to take into account both causal influence at multiple levels as well as the dynamic and interconnected relationships between causal factors that lie between the exposure (schizophrenia) and the outcome (premature mortality) (Galea et al., 2010).

A causal loop diagram (CLD) is a systems thinking tool that illustrates the causal relationships among a set of factors operating in a system (Maani & Cavana, 2007). It enables empirical research and theoretical knowledge to be combined into a shared ‘big picture’ understanding of a particular problem. CLDs allow us to investigate non-linear causality, where the effects of a cause can be traced back through a set of variables to the original cause, thus creating a feedback loop. Feedback loops are the mechanisms that generate the behaviour of a system (Meadows, 2009). There are two types of feedback processes: reinforcing and balancing. Reinforcing feedback is self-enhancing, reinforcing whatever direction of change is imposed on the system, resulting in a virtuous or vicious cycle, that is favourable or detrimental outcomes. Balancing feedback on the other hand is stability-seeking, opposing whatever direction of change is imposed on the system and thus maintaining equilibrium. Every feedback loop tells a story and can help explain why we see particular behaviours persist over time. A CLD provides a conceptual framework that can help us interpret the current evidence base, identify potential intervention points and highlight areas in need of further research.

The aim of this study is to better understand why the mortality gap between people with schizophrenia and the general population is not improving – what is stopping the life expectancy of people with schizophrenia from reaching that of the general population? This will be achieved by developing a CLD based on published literature and consultation with experts. We were guided by the following research questions outlined by Petticrew et al. (2019): What are the main influences on the health problem? How are they created and maintained? How are they interconnected? Where might one intervene in the system?

## Methods

### Defining the boundaries

In terms of defining the boundaries of the system, the focus of the CLD was what happens after a person develops schizophrenia and potentially modifiable factors. This excluded biological factors and early life exposures that may contribute to the development of both schizophrenia and physical diseases. Within the CLD, premature mortality was defined as death that occurs before the average life expectancy of a particular population. To keep the scope within the research question (what is stopping life expectancy from improving), research on interventions (looking at what would improve life expectancy) was not included. Comorbid mental disorder diagnoses were also excluded to keep the focus on schizophrenia specifically.

### Using literature to identify key variables and relationships

An initial version of the CLD was drafted drawing on Liu *et al.*’s multilevel model of risk for excess mortality in people with severe mental disorders (SMDs), with variables classified at the individual, healthcare and social levels (Liu et al., 2017). Literature was then used to substantiate causal relationships and identify other relevant variables. This initially consisted of articles that had been collected during the process of a previous systematic review quantifying excess mortality in SMDs (Ali et al., 2022). These articles were mostly those excluded during the title-abstract screening, as they were not eligible for the quantitative synthesis but did explore factors mediating the relationship between SMDs and mortality. Firstly, systematic reviews and meta-analyses were used to identify well-established relationships. Single studies were then used to further explore specific variables and relationships. Both quantitative and qualitative evidence were included. Narrative reviews were also drawn upon, particularly to incorporate relationships which appear important but for which empirical evidence is lacking. Data was extracted into Microsoft Excel spreadsheets designed for import into Kumu, the platform used to create the CLD (Kumu, 2022). Details of the extraction spreadsheets are provided in the supplementary methods.

### Refining the CLD and identifying feedback loops

The conceptualisation of variables was refined as data extraction progressed, for example, ‘hospitalisation’ was replaced by ‘acute illness’ in order to provide a clearer link to the ‘premature mortality’ variable; ‘regular care’ was replaced by the term ‘quality of care’ to include issues around clinical guidelines not being followed and poor relationships between health professionals and patients.

For each connection, possible mediators between variables were also considered. Feedback loops were identified by taking each variable and following consecutive links until the variable was reached again, as well as through the automatic loop detection feature in Kumu. Study authors critically reflected on each loop to ensure the underlying logic was consistent with the expected behaviour of the system, and also aligned with the evidence in the connections, for example, a loop could be created using metabolic abnormalities to stigma to quality of care, however, metabolic abnormalities results in self-stigma, which is different from the perceived or experienced stigma from health professional which reduces quality of care. Hypothesised connections and variables that arose from the process of identifying and reviewing loops were substantiated if possible using the literature, with additional articles sought out if necessary. The variables, connections and feedback loops were iteratively reviewed and refined until they best reflected the available literature and the study authors’ knowledge and experience.

### Expert consultation

A consultation was held with Australian researchers with relevant expertise in order to gain feedback on the CLD that could be used to further refine the model. Participants were selected based on their expertise in the topic of premature mortality and physical health in people with schizophrenia or clinical experience working with people with this disorder. Four participants were invited, with backgrounds in psychiatry, mental health nursing and primary care; three agreed to participate. The consultation session was held online, with the participants asked to review the interactive CLD beforehand. They were asked to consider if the variables, connections and feedback loops aligned with their professional knowledge and experience; if they could further substantiate any connections, particularly those based on less evidence; if they agreed with how the variables were defined, in terms of level of detail and specificity; and if there were any important variables or connections that they thought were missing. The feedback obtained was used to more clearly define the boundaries of the model, revise the definition of variables as well as add perspectives to the discussion. Additionally, a subsystem diagram was created to provide a simplified overview of the processes which the CLD explores in detail.

### CLD principles and notation

The basic elements of a CLD are variables and directional arrows, or connections. These connections represent causal relationships and there are two types:

- Positive – denoted by a +, where a change in a variable causes a change in the variable it connects to in the *same* direction, that is an increase causes a corresponding increase
- Negative – denoted by a -, where a change in a variable causes a change in the variable it connects to in the *opposite* direction, that is an increase causes a corresponding decrease

There are also two types of feedback loops:

- Reinforcing – denoted by a capital R, where changes in any variable are propagated through the loop to reinforce the initial change; contains an even number of negative connections
- Balancing – denoted by a capital B, where the loop structure limits changes in variables and stability is maintained; contains an odd number of negative connections

### Ethics approval

The study was approved by the University of Queensland Human Ethics Committee (project number: 2022/HE001615).

## Results

### Subsystem diagram

**Figure 1.**
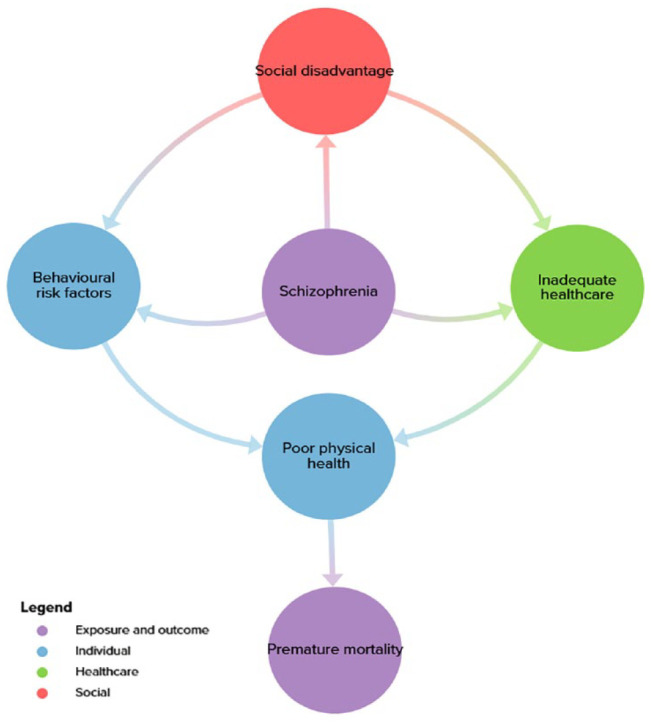
Subsystem diagram of the overall system structure of premature mortality from comorbid physical diseases in people with schizophrenia.

Figure 1 provides a high-level overview of the system which results in premature mortality from comorbid physical diseases in people with schizophrenia. The diagram illustrates how poor health outcomes are primarily mediated through behavioural risk factors and inadequate healthcare, which in turn are influenced by social disadvantage.

### Overall CLD

**Figure 2.**
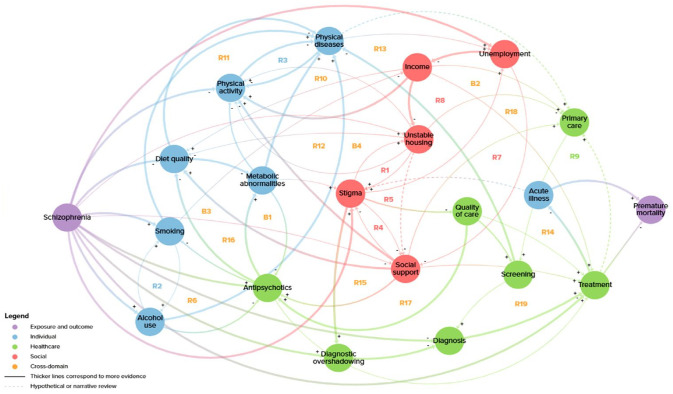
Causal loop diagram of premature mortality from comorbid physical diseases in people with schizophrenia.

The CLD shown in Figure 2 represents the causal relationships and feedback processes that are driving the mortality gap between people with schizophrenia and the general population. An interactive, web-based version of the CLD is available at: https://suhailaha.kumu.io/schizophrenia-and-premature-mortality?token=79S31Be5KJejpFbK. A total of 74 references were included as supporting evidence for variables and connections, and were categorised as systematic reviews, meta-analyses, narrative reviews, cohort studies, cross-sectional studies, longitudinal studies, case-control studies and qualitative studies (Supplemental Tables S1 and S2). Connections could also be based on expert opinion, hypothesised or categorised as established if the relationship was readily apparent and did not need a specific reference provided, for example, unemployment reducing income. The style and thickness of the lines in the CLD correspond to the amount of evidence the connection is based on: dashed – hypothesised or derived from narrative reviews; thin – one study; medium – multiple studies; and thick – systematic reviews and meta-analyses, or established.

A total of 21 variables were included in the CLD, with schizophrenia (the exposure) positioned as an exogenous variable, representing the initial conditions of the system and influencing but not being influenced by other variables (Supplemental Table S1). The remaining variables were endogenous, both influencing and influenced by other variables and therefore involved in feedback loops, except for premature mortality (the outcome) which was only influenced by other variables. Table 1 shows the variables ranked by the number of connections. There were a total of 68 connections in the CLD, and the variables with the most connections were stigma and social support (10 each), while diagnostic overshadowing and acute illness had the least connections (3 each) (Supplemental Table S2).

**Table 1.** Total number of connections and feedback loops for each variable.

Variable	Total connections (n)	Total feedback loops (n)
Stigma	10	8
Social support	10	6
Physical diseases	9	5
Antipsychotics	9	6
Treatment	9	4
Unstable housing	8	4
Income	7	5
Physical activity	7	3
Diet quality	7	3
Metabolic abnormalities	6	2
Primary care	6	3
Smoking	6	4
Alcohol use	5	2
Unemployment	5	10
Diagnosis	4	3
Quality of care	4	2
Screening	4	4
Diagnostic overshadowing	3	1
Acute illness	3	3

### Feedback loops

A total of 23 distinct feedback loops were identified, including 19 reinforcing loops and 4 balancing loops (Supplemental Table S3). The majority were cross-domain loops, alongside five loops operating entirely within the social domain, two within the individual domain and one in the healthcare domain. Unemployment was involved in the greatest number of loops (10), followed by stigma (8); while diagnostic overshadowing was involved in the fewest loops (1), followed by metabolic abnormalities, alcohol use and quality of care, all of which were involved in two loops each (Table 1). The moderators listed in Supplemental Table S3 refer to variables that could be added to the referenced loop. For example, for R8, social support could be added in between stigma and unstable housing. These were highlighted in the table to capture the additional interactions but not labelled as separate loops since the main mechanism is represented in the primary loop. Note that in the figures below, each loop can be traced in Figure 2 and the interactive CLD but some positions have been rearranged for readability.

**Figure 3.**
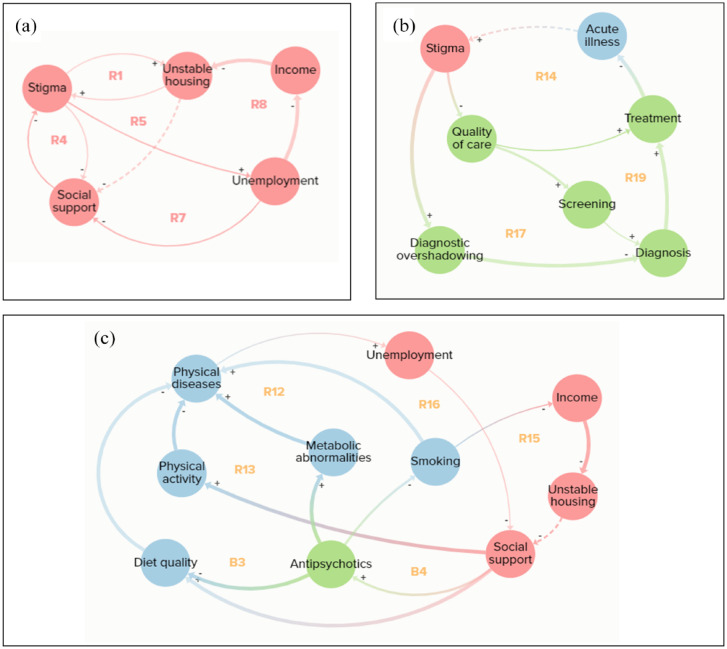
(a) Social domain feedback loops, (b) stigma and healthcare feedback loops and (c) social support feedback loops.

The five loops operating within the social domain all exhibited reinforcing feedback behaviour (Figure 3a). This demonstrates how factors related to social disadvantage act to perpetuate each other, with a central role of stigma, which contributed to unstable housing, reduced social support and unemployment, all of which then acted to reinforce stigma. Besides the two established connections from unemployment to income to unstable housing, most connections shown in Figure 3a are based on single studies and narrative reviews.

**Figure 4.**
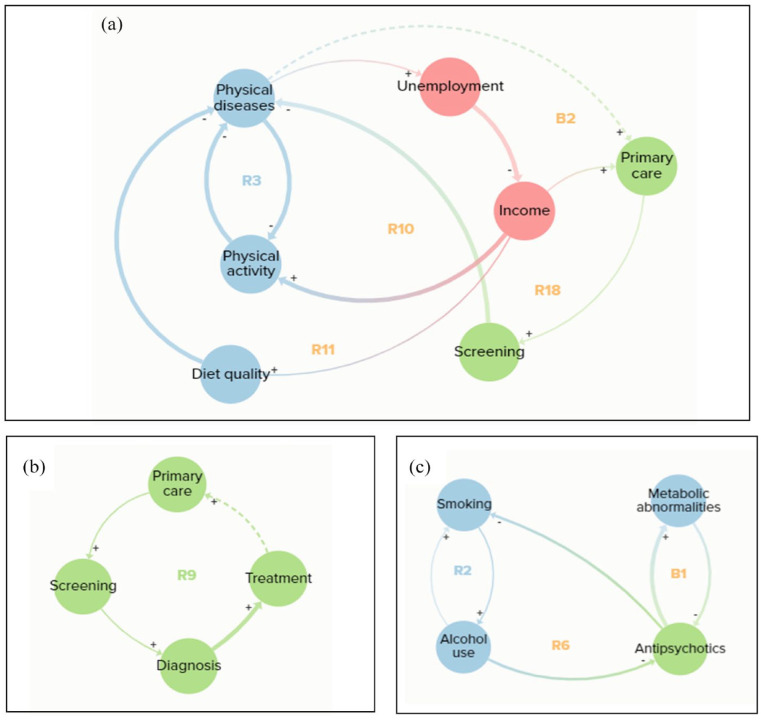
(a) Comorbidity and income feedback loops, (b) healthcare domain feedback loop and (c) smoking, alcohol and medication feedback loops.

There were several common pathways that appeared in more than one feedback loop. These were treatment to acute illness to stigma (Figure 3b); physical diseases to unemployment to social support (Figure 3c); social support to antipsychotics to smoking (Figure 3c) and physical diseases to unemployment to income (Figure 4a).

Figure 3b demonstrates the multiple mechanisms through which stigma operates in healthcare pathways: it reduces quality of care, leading to treatments not being offered even when physical health issues are identified (R14); reduced quality of care also decreases screening, which reduces diagnosis and therefore treatment (R19); stigma also leads to diagnostic overshadowing, again reducing diagnosis and treatment (R17). This lack of treatment results in patients developing acute issues, which may reinforce stigma in healthcare providers around patients not being interested in their physical health and healthcare.

Figure 3c illustrates the importance of social support, without which it becomes difficult to have a healthy diet (R12) and take part in physical activity (R13) which both contribute to physical disease comorbidity, making it hard to work and further reducing access to social support. There are also two balancing loops where taking antipsychotics contributes to poor diet quality (B3) and metabolic abnormalities (B4), again leading to comorbidities, unemployment and reduced social support, which subsequently reduces antipsychotic adherence. This reduction in medication adherence also interferes with smoking cessation, which again acts to reduce social support through the comorbidity-unemployment pathway (R16). Smoking also reduces income, contributing to unstable housing and further disrupting social support (R15).

Figure 4a demonstrates how comorbidities can affect individual risk factors, either directly in terms of making it difficult to exercise (R3) or through unemployment which reduces income, with cost being a barrier to physical activity (R10) and healthy food (R11), thus contributing further to poor physical health. Reduced income also hinders primary care access, which then reduces screening, making it more likely for patients to develop comorbidities (R18). There is also a balancing loop where physical diseases may lead to increased use of primary care, providing more opportunities for screening and monitoring of physical health, which can prevent diseases from getting worse and the development of additional comorbidities (B2).

The single loop that operated only within the healthcare domain represented a virtuous cycle, where primary care enhances screening, leading to diagnosis and treatment, and a hypothesised connection that with successful treatment, patients are likely to access primary care again for ongoing monitoring and if new problems arise (R9; Figure 4b).

Finally, Figure 4c demonstrates how smoking and alcohol reinforce each other (R2), with smoking also enhanced through alcohol reducing medication adherence (R6); adherence is also reduced due to the metabolic side effects of antipsychotics, resulting in a balancing loop (B1).

## Discussion

This is the first study to apply a systems thinking approach to the issue of premature mortality in people with schizophrenia. The CLD has provided a number of insights into our understanding of the problem, including influential factors and areas that need further research.

Stigma and social support had the most connections in the CLD, which demonstrates the important role of social determinants in shaping the health outcomes of people with schizophrenia. Out of the five social factors included in the CLD, there were five feedback loops. This shows the interconnected nature of social determinants and how they reinforce each other, making it difficult for someone experiencing social disadvantage to improve their circumstances without external changes, that is shifts in public policy and social norms (Shim & Compton, 2020). While in their current state these reinforcing cycles are producing poor outcomes, if even one of the factors in these loops is turned around, the cycle can be shifted to reinforce positive outcomes. Unemployment was involved in the most feedback loops and was also discussed in the expert consultation as being a factor that if targeted, would potentially be the most effective at improving outcomes. Looking at the CLD, we can see how employment would have a number of downstream effects, including better social support, reduced stigma and increased income leading to more stable housing, easier access to primary care and less behavioural risk factors. It is important to note that there was a lack of evidence around social determinants, in contrast to the large amount of evidence related to individual and healthcare factors, which reflects the dominant biomedical paradigm in which research priorities have predominantly existed.

There were only two feedback loops operating entirely within the individual domain, with the majority of loops involving healthcare and social factors. This is important as behavioural risk factors in particular are often framed as the responsibility of the individual and the result of their ‘poor choices’; the CLD demonstrates that we cannot ignore a person’s experiences in the health system and the social context in which they live. It is interesting to note that metabolic abnormalities were only involved in two feedback loops, both of which were balancing loops that served to reduce antipsychotic adherence. Metabolic abnormalities resulting from antipsychotics are frequently pointed to as a contributing factor to premature mortality, which appears to contrast with the fact that antipsychotics are in fact protective of mortality (Correll et al., 2022; Taipale et al., 2020). The CLD shows that metabolic abnormalities do not play a major role the system; while they do contribute to the development of physical diseases, in terms of system behaviour the main contribution was reducing medication adherence. Addressing these side effects would therefore not only reduce the risk of comorbidities, it would also increase antipsychotic compliance and the subsequent protective effects on mortality. These have yet to be fully elucidated, with suggestions that improved symptom control facilitates healthy behaviours and better engagement with the healthcare system (Correll et al., 2018). In the CLD, the only variable that acts to directly reduce premature mortality is treatment, which is increased by antipsychotics; this suggests that improved self-management and treatment may play an important role in the mortality risk-reducing effect of antipsychotics.

In terms of the healthcare system, stigma from health professionals was shown to play a central role, resulting in physical diseases not getting diagnosed and treated, leading to acute illness and potentially reinforcing stigma. This demonstrates the importance of relationships between health professionals and people with schizophrenia, with qualitative studies advocating for shared decision-making and person-centred care in order to build trust and make patients feel respected, and empowered to take steps to care for their physical health (Lerbaek et al., 2019; Ross et al., 2015; Stumbo et al., 2018). Qualitative research also showed that patients often prioritised their mental health over physical health, with health professionals similarly focussed on the mental disorder diagnosis but with stigma attached, viewing the disorder as a barrier to providing care (Balogun-Katung et al., 2021). Effective mental and physical healthcare requires shared responsibility and collaboration between mental health services and primary care providers, viewing patients as whole people with unique needs, not just a diagnostic label (Ross et al., 2015; Young et al., 2017). Improved quality of care would not only encourage better self-management but also facilitate continuity of care. Alongside addressing the social determinants that hinder primary care access, this would lead to a positive cycle where instead of visiting a GP for severe concerns, regular monitoring would detect risk factors and diseases early. A critical barrier to building better relationships and addressing complex issues in primary care is limited time, which could be improved by utilising a team-based model of care with allied health workers sharing the workload with GPs (Breadon, 2022). Additional support from allied health workers would also facilitate tailored preventative care and follow-up to coordinate ongoing care (Stumbo et al., 2018). This links in with holistic and integrated models of care that not only address clinical needs but also practical, social and financial needs (Rethink Mental Illness, 2022).

It should be noted that there were important factors excluded from the CLD due to the boundaries of the model and the scope of evidence from the systematic review. These included demographic characteristics such as race and ethnicity, which influence the risk of developing physical diseases, healthcare provision and social determinants of health (Misra et al., 2022; O’Gallagher et al., 2022; Shim & Compton, 2020); biological factors such as the gut microbiome which may influence certain outcomes during the course and treatment of the disorder (Vasileva et al., 2022); and commercial determinants of health that shape the environment in which people live and subsequently, their health-related behaviours, for example tobacco retailer densities (Kickbusch et al., 2016; Young-Wolff et al., 2014).

One of the strengths of systems thinking methodology is the ability to incorporate different types of evidence. Research in this topic has typically been dominated by quantitative studies and perspectives from psychiatrists. In this CLD, we were able to incorporative qualitative evidence, including studies looking at the lived experience of patients which is often overlooked. Another strength of CLDs is that they can be continually updated as new information emerges and with input from different stakeholders; for example, the CLD could be further refined in collaboration with people with schizophrenia, or contextualised with more evidence from low- and middle-income settings which is lacking in this area. Limitations to the study include using some studies that looked at different types of SMDs together, where different disorders can potentially vary in the associations they have with particular variables. Some cross-sectional studies were also used, which are unable to show the direction of causality between variables so further evidence, including longitudinal studies, would be needed to substantiate those associations.

In terms of future research, the next phase of systems thinking methodology after causal loop modelling is dynamic modelling, which involves developing a quantitative simulation model (Maani & Cavana, 2007). This phase involves collecting detailed data to then set values for the variables and relationships, which can be specific to a particular country and health system. The model can then be used to simulate the impact of different policies and practices over time and test potential interventions.

In conclusion, the CLD has created a holistic and dynamic understanding of the causal pathways driving the mortality gap between people with schizophrenia and the general population. Previous research has typically examined contributing factors in linear terms, which misses the complexities involved. Visualising the interactions between these factors has revealed underlying feedback mechanisms, providing insights into why the mortality gap has persisted over time. In particular, the CLD has highlighted the need to address social determinants of health, with further research on these factors required to build the evidence base for decision-making, broadening the focus of potential interventions outside of the clinical domain. The findings of this study align closely with the priorities for action outlined in the recently published *Gone Too Soon* framework, and help to address the gap highlighted by the authors in terms of understanding the mediating pathways between social determinants and premature mortality (O’Connor et al., 2023). The CLD also challenges the dominant narrative around the contribution of metabolic side effects of antipsychotics to premature mortality and emphasises the importance of medication adherence. Actions are needed within health systems, particularly to combat stigma, as well as at the government level, for example in terms of employment support, in order to reshape this system to produce better outcomes for people with schizophrenia.

## Supplemental Material

sj-docx-1-isp-10.1177_00207640231194477 – Supplemental material for Using a systems thinking approach to explore the complex relationships between schizophrenia and premature mortality Click here for additional data file. Supplemental material, sj-docx-1-isp-10.1177_00207640231194477 for Using a systems thinking approach to explore the complex relationships between schizophrenia and premature mortality by Suhailah Ali, Eryn Wright and Fiona Charlson in International Journal of Social Psychiatry

## References

[bibr1-00207640231194477] Ali S. Santomauro D. Ferrari A. J. Charlson F. (2022). Excess mortality in severe mental disorders: A systematic review and meta-regression. Journal of Psychiatric Research, 149, 97–105. 10.1016/j.jpsychires.2022.02.036 35259666

[bibr2-00207640231194477] Arnold R. D. Wade J. P. (2015). A definition of systems thinking: A systems approach. Procedia Computer Science, 44, 669–678. 10.1016/j.procs.2015.03.050

[bibr3-00207640231194477] Balogun-Katung A. Carswell C. Brown J. V. E. Coventry P. Ajjan R. Alderson S. Bellass S. Boehnke J. R. Holt R. Jacobs R. Kellar I. Kitchen C. Lister J. Peckham E. Shiers D. Siddiqi N. Wright J. Young B. Taylor J. team D. r. (2021). Exploring the facilitators, barriers, and strategies for self-management in adults living with severe mental illness, with and without long-term conditions: A qualitative evidence synthesis. PloS One, 16(10), e0258937. 10.1371/journal.pone.0258937 PMC8547651 34699536

[bibr4-00207640231194477] Breadon P. Romanes D. Fox L. Bolton J. Richardson L. (2022). A new medicare: Strengthening general practice. Grattan Institute.

[bibr5-00207640231194477] Cerda M. Keyes K. M. (2019). Systems modeling to advance the promise of data science in epidemiology. American Journal of Epidemiology, 188(5), 862–865. 10.1093/aje/kwy262 30877289 PMC6494667

[bibr6-00207640231194477] Correll C. U. Rubio J. M. Kane J. M. (2018). What is the risk-benefit ratio of long-term antipsychotic treatment in people with schizophrenia? World Psychiatry, 17(2), 149–160. 10.1002/wps.20516 29856543 PMC5980517

[bibr7-00207640231194477] Correll C. U. Solmi M. Croatto G. Schneider L. K. Rohani-Montez S. C. Fairley L. Smith N. Bitter I. Gorwood P. Taipale H. Tiihonen J. (2022). Mortality in people with schizophrenia: A systematic review and meta-analysis of relative risk and aggravating or attenuating factors. World Psychiatry, 21(2), 248–271. https://doi.org/https://doi.org/10.1002/wps.20994 35524619 10.1002/wps.20994 PMC9077617

[bibr8-00207640231194477] Crump C. Winkleby M. A. Sundquist K. Sundquist J. (2013). Comorbidities and mortality in persons with schizophrenia: A Swedish national cohort study. The American Journal of Psychiatry, 170(3), 324–333. 10.1176/appi.ajp.2012.12050599 23318474

[bibr9-00207640231194477] Galea S. Riddle M. Kaplan G. A. (2010). Causal thinking and complex system approaches in epidemiology. International Journal of Epidemiology, 39(1), 97–106. 10.1093/ije/dyp296 19820105 PMC2912489

[bibr10-00207640231194477] Hjorthøj C. Stürup A. E. McGrath J. J. Nordentoft M. (2017). Years of potential life lost and life expectancy in schizophrenia: A systematic review and meta-analysis. The Lancet Psychiatry, 4(4), 295–301. 10.1016/S2215-0366(17)30078-0 28237639

[bibr11-00207640231194477] Kickbusch I. Allen L. Franz C. (2016). The commercial determinants of health. Lancet Global Health, 4(12), e895–e896. 10.1016/S2214-109X(16)30217-0 27855860

[bibr12-00207640231194477] Kumu. (2022). Kumu relationship mapping software. https://kumu.io

[bibr13-00207640231194477] Laursen T. M. Plana-Ripoll O. Andersen P. K. McGrath J. J. Toender A. Nordentoft M. Canudas-Romo V. Erlangsen A. (2018). Cause-specific life years lost among persons diagnosed with schizophrenia: Is it getting better or worse? Schizophrenia Research, 206, 284–290. 10.1016/j.schres.2018.11.003 30446270

[bibr14-00207640231194477] Lerbaek B. Jorgensen R. Aagaard J. Nordgaard J. Buus N. (2019). Mental health care professionals’ accounts of actions and responsibilities related to managing physical health among people with severe mental illness. Archives of Psychiatric Nursing, 33(2), 174–181. 10.1016/j.apnu.2018.11.006 30927987

[bibr15-00207640231194477] Liu N. H. Daumit G. L. Dua T. Aquila R. Charlson F. Cuijpers P. Druss B. Dudek K. Freeman M. Fujii C. Gaebel W. Hegerl U. Levav I. Munk Laursen T. Ma H. Maj M. Elena Medina-Mora M. Nordentoft M. Prabhakaran D. , . . . Saxena S. (2017). Excess mortality in persons with severe mental disorders: A multilevel intervention framework and priorities for clinical practice, policy and research agendas. World Psychiatry, 16(1), 30-40. https://doi.org/doi:10.1002/wps.20384 28127922 10.1002/wps.20384 PMC5269481

[bibr16-00207640231194477] Maani K. Cavana R. Y. (2007). Systems thinking, system dynamics: Managing change and complexity (2nd ed.). Pearson Education New Zealand.

[bibr17-00207640231194477] Meadows D. H. (2009). Thinking in systems: A primer ( Wright D. , Ed.). Earthscan.

[bibr18-00207640231194477] Misra S. Etkins O. S. Yang L. H. Williams D. R. (2022). Structural racism and inequities in incidence, course of illness, and treatment of psychotic disorders among black Americans. American Journal of Public Health, 112(4), 624–632. 10.2105/ajph.2021.306631 35319958 PMC8961835

[bibr19-00207640231194477] O’Connor R. C. Worthman C. M. Abanga M. Athanassopoulou N. Boyce N. Chan L. F. Christensen H. Das-Munshi J. Downs J. Koenen K. C. Moutier C. Y. Templeton P. Batterham P. Brakspear K. Frank R. G. Gilbody S. Gureje O. Henderson D. John A. , . . . Yip P. S. F. (2023). Gone Too Soon: priorities for action to prevent premature mortality associated with mental illness and mental distress. Lancet Psychiatry, 10(6), 452-464. 10.1016/S2215-0366(23)00058-5 37182526

[bibr20-00207640231194477] O’Gallagher K. Teo J. T. Shah A. M. Gaughran F. (2022). Interaction between race, ethnicity, severe mental illness, and cardiovascular disease. Journal of American Heart Association, 11(12), e025621. 10.1161/JAHA.121.025621 PMC9238657 35699192

[bibr21-00207640231194477] Peters D. H. (2014). The application of systems thinking in health: Why use systems thinking? Health Research Policy and Systems, 12, 51. 10.1186/1478-4505-12-51 25160707 PMC4245196

[bibr22-00207640231194477] Petticrew M. Knai C. Thomas J. Rehfuess E. A. Noyes J. Gerhardus A. Grimshaw J. M. Rutter H. McGill E. (2019). Implications of a complexity perspective for systematic reviews and guideline development in health decision making. BMJ Global Health, 4(Suppl 1), e000899. 10.1136/bmjgh-2018-000899 PMC6350708 30775017

[bibr23-00207640231194477] Rethink Mental Illness. (2022). Building communities that care: A blueprint for supporting people severely affected by mental illness in their local communities by 2024. https://www.rethink.org/media/2249/building-communities-that-care-report.pdf

[bibr24-00207640231194477] Ross L. E. Vigod S. Wishart J. Waese M. Spence J. D. Oliver J. Chambers J. Anderson S. Shields R. (2015). Barriers and facilitators to primary care for people with mental health and/or substance use issues: A qualitative study. BMC Family Practice, 16, 135. 10.1186/s12875-015-0353-3 26463083 PMC4604001

[bibr25-00207640231194477] Shim R. S. Compton M. T. (2020). The social determinants of mental health: Psychiatrists’ roles in addressing discrimination and food insecurity. Focus (American Psychiatric Publishing), 18(1), 25–30. 10.1176/appi.focus.20190035 32047394 PMC7011221

[bibr26-00207640231194477] Stumbo S. P. Yarborough B. J. H. Yarborough M. T. Green C. A. (2018). Perspectives on providing and receiving preventive health care from primary care providers and their patients with mental illnesses. American Journal of Health Promotion, 32(8), 1730–1739. 10.1177/0890117118763233 29658287 PMC7220499

[bibr27-00207640231194477] Taipale H. Tanskanen A. Mehtälä J. Vattulainen P. Correll C. U. Tiihonen J. (2020). 20-year follow-up study of physical morbidity and mortality in relationship to antipsychotic treatment in a nationwide cohort of 62,250 patients with schizophrenia (FIN20). World Psychiatry, 19(1), 61–68. 10.1002/wps.20699 31922669 PMC6953552

[bibr28-00207640231194477] van Zonneveld S. M. Haarman B. C. M. van den Oever E. J. Nuninga J. O. Sommer I. E. C. (2022). Unhealthy diet in schizophrenia spectrum disorders. Current Opinion Psychiatry, 35(3), 177–185. 10.1097/YCO.0000000000000791 35585755

[bibr29-00207640231194477] Vasileva S. S. Tucker J. Siskind D. Eyles D. (2022). Does the gut microbiome mediate antipsychotic-induced metabolic side effects in schizophrenia? Expert Opinion on Drug Safety, 21, 625–639. 10.1080/14740338.2022.2042251 35189774

[bibr30-00207640231194477] Young S. J. Praskova A. Hayward N. Patterson S. (2017). Attending to physical health in mental health services in Australia: A qualitative study of service users’ experiences and expectations. Health & Social Care in the Community, 25(2), 602–611. 10.1111/hsc.12349 27093882

[bibr31-00207640231194477] Young-Wolff K. C. Henriksen L. Delucchi K. Prochaska J. J. (2014). Tobacco retailer proximity and density and nicotine dependence among smokers with serious mental illness. American Journal of Public Health, 104(8), 1454–1463. 10.2105/AJPH.2014.301917 24922145 PMC4103201

