# Supplementary material for: Using a systems thinking approach to explore the complex relationships between schizophrenia and premature mortality
Source: Int J Soc Psychiatry. 2023 Aug 29;70(1):70–9. doi: 10.1177/00207640231194477 (PMC10860352; doi:10.1177/00207640231194477)
Supplement: sj-docx-1-isp-10.1177_00207640231194477 – Supplemental material for Using a systems thinking approach to explore the complex relationships between schizophrenia and premature mortality [file sj-docx-1-isp-10.1177_00207640231194477.docx]

**Supplementary material**

*Supplementary methods*

The extraction spreadsheet consisted of an ‘Elements’ sheet, which included the names of each variable, type (individual, healthcare, social), descriptions, time trends (if applicable) and references; and a ‘Connections’ sheet which included ‘From’ and ‘To’ columns to capture relationships between variables, type to indicate polarity (+ for positive associations, - for negative associations), tags to specify study type (e.g., meta-analysis, systematic review, cohort study), descriptions and references.

**Table S1: Variables included in causal loop diagram**

| **Variable** | **Domain** | **Description** | **Time trends** | **References** |
| --- | --- | --- | --- | --- |
| Schizophrenia | Exposure | The clinical features that constitute schizophrenia include positive symptoms, also known as psychosis or the psychotic syndrome (i.e., delusions, hallucinations, and formal thought disorder [speech that is difficult to follow, sometimes to the point of incomprehensibility]); negative symptoms, which consist of lack of volition, reduced speech output, and flattening of affect (i.e., decreased expression of emotions); and cognitive impairment, where patients commonly show poor performance on tests of executive function, long-term memory, and sustained attention, as well as a variable degree of general intellectual impairment. | The number of schizophrenia cases globally has increased from 14.2 million in 1990 to 23.6 million in 2019; the age-standardised prevalence per 100,000 people has decreased slightly from 289.9 in 1990 to 287.4 in 2019. | (GBD 2019 Mental Disorders Collaborators, 2022; Jauhar et al., 2022) |
| Premature mortality | Outcome | Death that occurs before the average life expectancy of a particular population | Mixed findings around all-cause mortality risk over time; some meta-analyses have show increasing risk, others have shown no significant changes. Mortality due to natural causes appears to be increasing, while unnatural mortality remains stable. | (Ali et al., 2022) |
| Acute illness | Individual | Experiencing severe stages of a physical disease, developing complications; likely to be hospitalised |  |  |
| Alcohol use | Individual | Alcohol use disorder; alcohol abuse/dependence | From 2009 review - when we compared our results with that of studies published between 1960–1989 and 1990–1995, our median prevalence in lifetime abuse was statistically significantly lower. This change may mainly be due to variation in diagnostics, sampling and other methodological differences, but it may also indicate a descending trend in alcohol use disorders (AUD) in patients with schizophrenia. From 2018 review - prevalence of AUD in people with schizophrenia did not differ over five time blocks (1984–1994, 1995–1999, 2000–2004, 2005–2009 and 2010–2017). | (Hunt et al., 2018; Koskinen et al., 2009) |
| Physical activity | Individual | Includes exercise and incidental activity (e.g., active transport); key physical activity guidelines for patients with schizophrenia state that physical inactivity should be avoided, with patients who participate in any amount of physical activity gaining some health benefits |  | (Vancampfort, De Hert, et al., 2012) |
| Metabolic abnormalities | Individual | Includes weight gain/high BMI, high fasting plasma glucose, high LDL cholesterol, high systolic blood pressure |  |  |
| Physical diseases | Individual | Comorbid physical health conditions |  |  |
| Diet quality | Individual | Encompasses protective diet components including fruit, vegetables, whole grains, fibre and legumes; and harmful diet components including processed meat, sugar-sweetened beverages, trans fatty acids and sodium; as well as dietary energy intake. |  | (GBD 2019 Risk Factors Collaborators, 2020) |
| Smoking | Individual | Tobacco smoking | In a US-based study looking at cigarette smoking in people severe mental illness compared to people without a psychiatric disorder, the prevalence of smoking in the sample as a whole decreased significantly over the study period (1999-2016), consistent with trends in the general U.S. population; however, the decrease was largely attributable to the control group. There were no significant time trends found in the groups with psychiatric diagnoses (including schizophrenia). | (Dickerson et al., 2018) |
| Antipsychotics | Healthcare | Antipsychotics are a type of psychotropic medication primarily used to managed psychosis. Standard management of schizophrenia includes the use of antipsychotic medications to help control acute psychotic episodes and prevent relapses, with maintenance therapy used in the long term after patients have been stabilized. Two main classes of drugs—first- and second-generation antipsychotics (FGA and SGA)—are used to treat schizophrenia. | FGAs introduced in 1950s; contributed to deinstitutionalisation, reduced hospitalisation rates, however, also caused extrapyramidal symptoms (EPS). Clozapine reintroduced in the 1990s, led to development of other SGAs; reduced risk of EPS but increased metabolic side effects. | (Correll et al., 2022; Solmi et al., 2017) |
| Diagnosis | Healthcare | Diagnosis of physical diseases |  |  |
| Diagnostic overshadowing | Healthcare | Physical symptoms are misattributed to mental disorders by health professionals |  |  |
| Quality of care | Healthcare | The degree to which health services increase the likelihood of desired health outcomes; encompasses evidence-based care, following clinical guidelines, continuity of care, patient-clinician relationships |  |  |
| Primary care | Healthcare | Primary care refers to the professional health care provided in the community, usually from a general practitioner (GP), nurse, pharmacist or allied health professional; primary care covers a range of services, including diagnosis and treatment, health promotion and prevention, and managing long-term conditions | Primary care consultation rates for people with severe mental illness increased over time (observation period 2000-2012); the increase was greater after the introduction of the Quality and Outcomes Framework (QOF) in 2004 [UK-specific]. | (Kontopantelis et al., 2015) |
| Screening | Healthcare | This includes both routine monitoring of physical health as well as population-based screening tests, which aim to detect people at higher risk of a particular disease e.g. cervical cancer screening | Based on the Survey of High Impact Psychosis (Australia), the proportion of people with a psychotic illness who received a physical health examination or a fasting blood test over a 12-month period had fallen; the percentages in 2010 were 66.1% for a physical health examination and 64.8% for a blood test compared to 79.6% and 83.1%, respectively, in Low Prevalence (Psychotic) Disorders Study in 1997–1998. This decline may reflect the transition of service provision from inpatient care (with mandatory examinisations as part of admission procedure) to community mental health care, with a reduction in the percentage of people with at least one psychiatric inpatient admission between the two surveys from 62.9% to 45.6%. | (Morgan et al., 2016) |
| Treatment | Healthcare | Treatments for physical diseases and risk factors e.g. cardiometabolic medications, surgical procedures | The gap in the use of coronary procedures, guideline-based therapy, and all-cause mortality following acute coronary syndrome in patients with schizophrenia compared to those without has remained constant over the past 2 decades [1996-2015]. In analysis of a large national inpatient database over the period of 2003 to 2012, SMI was persistently associated with reduced revascularization procedures compared to patients without SMI, despite a trend toward increased percutaneous revascularization reflecting changes in the management of ST-elevation myocardial infarction. | (Attar et al., 2020; Schulman-Marcus et al., 2016) |
| Income | Social | Includes wages and government payments |  |  |
| Social support | Social | Perceived and actual availability of functional support from an individual's social network (e.g. family, friends, colleagues, support groups), which can include emotional support, informational support (e.g. advice) and practical/tangible support (e.g. material assistance) |  |  |
| Stigma | Social | Stigma can be defined as the co-occurence of labelling, stereotyping, separation, status loss and discrimination; here we are specifically focussed on personal stigma (rather than public stigma, where the general population endorses prejudice and manifests discrimination toward people with mental illness), which consists of: perceived stigma - the perception or anticipation of stigma refers to people’s beliefs about attitudes of the general population towards their condition and towards themselves as members of a potentially stigmatized group; experienced stigma - discrimination or restrictions actually met by the affected persons; self-stigma - the internalization and adoption of stereotypic or stigmatizing views, i.e., of public stigma, by the stigmatized individual |  | (Gerlinger et al., 2013) |
| Unemployment | Social | Not in paid employment | Survey of High Impact Psychosis (Australia, 2010) found that 21.5% of participants were employed at the time of interview, and 32.7% had been employed at some time in the preceding 12 months. The percentage in employment had increased since the 1997–1998 Low Prevalence (Psychotic) Disorders Study, but was still disappointingly low given national initiatives over that period to improve disability employment services and the growing shift to a recovery-focussed framework for mental health service provision. | (Morgan et al., 2016) |
| Unstable housing | Social | Inadequacy of dwelling; lack of tenure; lack of control of, or access to, space in a living environment; overcrowding; moving frequently; trouble paying rent | Survey of High Impact Psychosis (Australia, 2010) found that housing circumstances since the first survey in 1997-1998 had improved; increased proportion of people living in rented accommodation (from 34.2% to 49.2%) and supported group housing (from 5.2% to 10.9%) and a drop in homelessness (from 13.0% to 5.0%) - likely to be linked with mental health service delivery changes such as deinstitutionalisation, and the further development of community-based mental health services, as well as broader policy developments that have targeted homelessness and the increased provision of affordable housing and support services for people with severe mental illness. | (Harvey et al., 2012) |

**Table S2: Connections included in causal loop diagram**

| **From** | **To** | **Type** | **Tags** | **Description** | **References** |
| --- | --- | --- | --- | --- | --- |
| Acute illness | Stigma | + | Hypothetical | Having acute physical health issues adds to the stigma around patients not being interested in their physical health and healthcare. |  |
| Acute illness | Premature mortality | + | Established | More severe stages of a physical disease can result in death |  |
| Alcohol use | Smoking | + | Cross-sectional study | Tobacco smoking associated with alcohol dependence in study of 474 patients with schizophrenia; explained at least by cross reinforcement, alcohol and nicotine potentiating each other’s rewarding effects. Future studies detailing the reciprocal impact of tobacco and alcohol in schizophrenia would be of interest. | (Mallet et al., 2019) |
| Alcohol use | Physical diseases | + | Meta-analysis | Contributes to digestive diseases, cancers, cardiovascular disease and respiratory infections | (GBD 2019 Risk Factors Collaborators, 2020) for data visualisations, visit <https://vizhub.healthdata.org/gbd-compare> |
| Alcohol use | Antipsychotics | - | Longitudinal study \| Case-control study \| Cross-sectional study | In a study using health insurance claims for 29,607 patients - compared to patients with schizophrenia who were medication adherent, patients with a stop-start pattern were more likely to have a history of alcohol abuse (odds ratio = 1.34; 95% confidence interval = 1.14-1.53), as well as patients in the immediate discontinuation group. Patients (n = 1598) with first-episode schizophrenia and comorbid alcohol use disorder (AUD) had lower medication adherence compared to a control group before and after diagnosis of AUD, also shorter mean total duration of antipsychotic treatment and higher number of treatment discontinuations. In a cross-sectional study of 154 patients with schizophrenia, alcohol was associated with worse adherence to medication. | (Ahn et al., 2021; Jonsdottir et al., 2013; MacEwan et al., 2016) |
| Antipsychotics | Smoking | - | Cohort study | Both typical antipsychotics and clozapine have been shown to enhance smoking reduction in patients with schizophrenia. | (Wu et al., 2013; Wu & Lan, 2017) |
| Antipsychotics | Diet quality | - | Systematic review | Clinical and animal study data suggest that increased appetite and food intake, as well as delayed satiety signaling, are key behavioral changes leading to antipsychotic-induced weight gain/obesity (via hormone and neurotransmitter interactions). | (Correll et al., 2015) |
| Antipsychotics | Metabolic abnormalities | + | Meta-analysis \| Narrative review | Antipsychotics were found to vary markedly in their effects on body weight, BMI, total cholesterol, LDL cholesterol, HDL cholesterol, triglycerides, and glucose concentrations; [...] clozapine and olanzapine [both second generation antipsychotics] are, across virtually all parameters, associated with the largest degree of metabolic dysregulation. Antipsychotic-induced metabolic alternations operate through a range of mechanisms, including impairments in lipid, carbohydrate and energy homeostasis which have direct metabolic consequences. | (Pillinger et al., 2020; Singh et al., 2019) |
| Antipsychotics | Treatment | + | Cohort study | Real-world evidence from 52,607 patients with schizophrenia prescribed statins, anti-diabetic medications, antihypertensives, and beta-blockers shows that current antipsychotic use is associated with a decreased risk of discontinuation of these medications compared with no antipsychotic use, accounting for subject-related characteristics using within-subject study design. Antipsychotics ranking best for discontinuation of all cardiometabolic drug categories were clozapine (adjusted hazard ratio [aHR] range = 0.34–0.55), followed by olanzapine (aHR = 0.43–0.71). | (M. Solmi et al., 2021) |
| Diagnosis | Treatment | + | Established | Once diagnosed with a disease, treatment can be provided |  |
| Diagnostic overshadowing | Diagnosis | - | Systematic review \| Qualitative study | From systematic review of qualitative studies - mental illness diagnosis dominated discussion, even if patients specifically presented to emergency department seeking help for physical illness symptoms; "by saying that there is a mental health problem, people overlook the physical health". From qualitative study - case described by member of consumer network where someone died of heart disease because doctor dismissed symptoms as part of their mental illness, so it went undiagnosed; another case of delayed diagnosis where patient's psychotropic medication was increased in response to physical symptoms, later diagnosed with diabetes when seen by a different provider. | (Happell et al., 2016; Molloy et al., 2021) |
| Diet quality | Metabolic abnormalities | - | Systematic review | Subjects with a poor diet and an unhealthy lifestyle were more likely to be overweight or obese, with high LDL cholesterol and low HDL cholesterol along with increased fasting glucose; a poor diet represents one of the factors involved in the development of metabolic abnormalities. | (Dipasquale et al., 2013) |
| Diet quality | Physical diseases | - | Meta-analysis | Poor diet quality contributes to cardiovascular disease, diabetes, kidney diseases and cancers | (GBD 2019 Risk Factors Collaborators, 2020) for data visualisations, visit <https://vizhub.healthdata.org/gbd-compare> |
| Income | Diet quality | + | Narrative review | Schizophrenia spectrum disorder patients have relatively low incomes. In Western countries, unhealthy food products are easily available and tend to be much cheaper than healthy ones, which facilitates the purchase of unhealthy food products by low-income groups. | (van Zonneveld et al., 2022) |
| Income | Primary care | + | Qualitative study | The majority of client participants in the study were living on disability or other income support, and as such, had very minimal incomes to cover basic necessities (e.g. housing and food) - healthcare not a priority relative to these more acute issues. Also lack of funds to cover costs of transportation to visit a primary care provider identified as a barrier. | (Ross et al., 2015) |
| Income | Unstable housing | - | Established | Securing and maintaining housing requires sufficient income; difficult to afford increasing cost of rent on a low income. |  |
| Income | Physical activity | + | Systematic review | Several studies illustrated that ‘self-management is expensive and resource intensive’; in terms of physical activity, participants in these studies described how they could not afford gym membership, clothes and equipment. | (Balogun-Katung et al., 2021) |
| Income | Treatment | + | Qualitative study | Economic constraints were identified by the respondents as a major factor in their ability to successfully manage their illnesses: the cost of diabetic care was frequently discussed; lack of money to pay for prescriptions compromised adherence to treatment for many respondents. | (El-Mallakh, 2007) |
| Metabolic abnormalities | Stigma | + | Qualitative study | Medication side effects including weight gain were experienced as stigmatising; these visible signs of the illness (or of the fact that one receives psychiatric treatment) connect the person displaying them with the negative stereotypes associated with mental illness. | (Schulze & Angermeyer, 2003) |
| Metabolic abnormalities | Physical activity | - | Qualitative study \| Cross-sectional study | In focus groups of outpatients with severe mental illness (mostly schizophrenia) looking at barriers to physical activity, the weight gain associated with many of the medications was a serious, long-term problem, with participants discussing how becoming obese had affected their physical activity - "It’s hard to exercise when you’re really overweight". In a cross-sectional study of 106 patients with schizophrenia, metabolic syndrome (MetS) was associated with poorer physical activity performance (compared to patients without MetS); patients with MetS also significantly less involved in sports activities and less physically active during leisure time. Qualitative review identified weight gain as side effect of medications as key barrier to engaging in physical activity. | (Balogun-Katung et al., 2021; McDevitt et al., 2006; Vancampfort et al., 2011) |
| Metabolic abnormalities | Antipsychotics | - | Cross-sectional study | From a survey of 304 people with schizophrenia, BMI status and subjective distress from weight gain were predictors of antipsychotic noncompliance. Obese individuals were more than twice as likely as those with a normal BMI to report missing their medication (odds ratio = 2.5; confidence interval 1.1–5.5). A comprehensive model suggested that the primary mediator of noncompliance was distress over weight gain. In a sample of 42 outpatients primarily with schizophrenia, 88% were overweight, 60% believed their medication contributed to their weight gain, of which 64% thought about stopping medication because of weight gain. | (Tham et al., 2009; Weiden et al., 2004) |
| Metabolic abnormalities | Physical diseases | + | Meta-analysis | Contributes primarily to cardiovascular disease, diabetes and kidney diseases | (GBD 2019 Risk Factors Collaborators, 2020) for data visualisations, visit <https://vizhub.healthdata.org/gbd-compare> |
| Physical activity | Physical diseases | - | Meta-analysis | Low physical activity contributes to cardiovascular disease, diabetes and kidney diseases | (GBD 2019 Risk Factors Collaborators, 2020) for data visualisations, visit <https://vizhub.healthdata.org/gbd-compare> |
| Physical diseases | Unemployment | + | Longitudinal study | In a longitudinal natural observation study of community residents (n = 104) with schizophrenia or schizoaffective disorder, comorbid physical health conditions were negatively associated with employment status. | (Waghorn et al., 2008) |
| Physical diseases | Physical activity | - | Systematic review | From systematic review of quantitative studies, the most consistent biological correlate of physical activity in people with schizophrenia was cardiometabolic comorbidity. Theme identified in systematic review of qualitative studies - ‘Physical health conditions limited people’s ability to engage in physical activity'; this resulted from symptoms such as chronic pain, difficulty breathing and fatigue. | (Balogun-Katung et al., 2021; Vancampfort, Knapen, et al., 2012) |
| Physical diseases | Primary care | + | Hypothetical | Systematic review showed severe mental illness (SMI) associated with increased primary care use compared to patients without SMI based on 7 studies with 10 analyses; authors suggest the primary reason for increased service use is likely the considerable rates of physical illness seen in patients with SMI. However, not able to rule out the use of primary care services for psychiatric reasons in any of the studies included in the review. | (Ronaldson et al., 2020) |
| Primary care | Screening | + | Longitudinal study | In a study using administrative claims data from 2010-2018, primary care utilisation was positively associated with cancer screening (for cervical, breast, colorectal and prostate cancer, odd ratios ranging from 2.13 to 4.51 compared to no primary care visits) among people with severe mental illness. | (Murphy et al., 2021) |
| Quality of care | Screening | + | Longitudinal study | In a study looking at breast cancer screening rates in women with schizophrenia, the strongest factor associated with screening was continuity of care (with general/family practioner), with good continuity associated with higher rates. Similar findings for cervical cancer screening; good continuity of care associated with higher screening rates. | (Chochinov et al., 2009; Martens et al., 2009) |
| Quality of care | Antipsychotics | + | Systematic review | The quality of the therapeutic relationship, as rated by both patients and clinicians, can indirectly influence adherence by mediating better attitudes to medication or to the psychiatric care in general. | (El Abdellati et al., 2020) |
| Quality of care | Treatment | + | Cross-sectional study | Examined the quality of assessment and treatment of physical health problems in people with schizophrenia (n = 5091) - most people with evidence of hypertension or dyslipidaemia had no record of being given appropriate treatment for these problems (25% and 20% offered intervention respectively). | (Crawford et al., 2014) |
| Schizophrenia | Smoking | + | Meta-analysis \| Longitudinal study | Prevalence of current smoking: 62%; effect size of current smoking, odds ratio: 5.3 (95% confidence interval 4.9 to 5.7). Evidence for a biological basis for increased nicotine use in schizophrenia, in terms of brain circuitry. | (de Leon & Diaz, 2005; Ward et al., 2022) |
| Schizophrenia | Alcohol use | + | Meta-analysis \| Narrative review | From 2009 review - prevalence of lifetime and current alcohol use disorder: 17.8% (interquartile ratio 9.7 to 28.6). From 2018 review - prevalence of alcohol use disorder: 24.3% (95% confidence interval 21.9 to 26.9). Based on epidemiological, genetic, brain imaging and pre-clinical studies, hypothesis put forward that the genetic determinants of risk for schizophrenia (especially within neural systems that contribute to the risk for both psychosis and addiction) make patients vulnerable to substance use. | (Hunt et al., 2018; Khokhar et al., 2018; Koskinen et al., 2009) |
| Schizophrenia | Physical activity | - | Meta-analysis \| Systematic review | Prevalence of low physical activity: 43.2% did not meet the recommended 150 min of moderate physical activity per week; people with schizophrenia engaged in significantly less moderate (hedges g: -0.45 [95% confidence interval (CI), -0.79 to -0.1]) and vigorous physical activity (hedges g: -0.39 [95% CI, -0.60 to -0.18]) compared to controls. Negative symptoms negatively associated with physical activity. Stress/depression most common barrier to exercise. | (Firth et al., 2016; Stubbs et al., 2016; Vancampfort, Knapen, et al., 2012) |
| Schizophrenia | Treatment | - | Meta-analysis \| Systematic review | Lower rates of treatment of any cardiovascular disease in people with schizophrenia compared to the general population; based on 27 studies, odds ratio = 0.597 (95% confidence interval 0.538 to 0.662). Qualitative synthesis found ‘severe mental illness symptoms’ were commonly reported to impact on people’s motivation and capacity for self-management and self-care generally. | (Balogun-Katung et al., 2021; Marco Solmi et al., 2021) |
| Schizophrenia | Diagnosis | - | Systematic review \| Qualitative study | Symptoms such as depression, paranoia, agoraphobia, agitation, anhedonia and social anxiety identified as barriers to attending appointments/getting out of the house; other barriers include crowded waiting rooms, waiting times; these barriers delay diagnosis. In primary care, patient-to-provider communication issues involved cultural misunderstandings, language barriers, difficulties in communicating as a result of symptoms, and lack of patient self-advocacy. Mental health care professionals also described how some people with severe mental illness were unable to articulate the symptoms of physical illness. | (Balogun-Katung et al., 2021; Kaufman et al., 2012; Lerbaek et al., 2019; Ross et al., 2015) |
| Schizophrenia | Diagnostic overshadowing | + | Systematic review | Mental health consumers felt uncertain about the origin of their illness experiences, with uncertainty further compounded by the physical illness exacerbating their mental illness symptoms and vice versa. | (Molloy et al., 2021) |
| Schizophrenia | Stigma | + | Systematic review | Based on 54 studies (n = 55,871), published from 1994 to 2011, on average, 64.5% (range: 45.0–80.0%) of patients with schizophrenia spectrum disorders perceived stigma, 55.9% (range: 22.5–96.0%) actually experienced stigma, and 49.2% (range: 27.9–77.0%) reported alienation (shame) as the most common aspect of self-stigma. | (Gerlinger et al., 2013) |
| Schizophrenia | Unemployment | + | Cohort study \| Systematic review | From Danish cohort study; at the age of 25, 87% of the individuals with schizophrenia were not employed, and at the age of 60, 85% of the individuals with schizophrenia were not employed; odds ratios (ORs) increased from 17.5 (95% confidence interval [CI] 16.4–18.6) to 48.9 (95% CI 44.1–54.3) between ages 25 and 45, and then decreased (at the age of 60: OR = 20.7, 95% CI 14.5–29.5); authors suggest cognitive and social functioning deficits as possible expanations. From Swedish cohort study: three years before first diagnosis, 24% of individuals with schizophrenia were employed; employment rate dropped around the time of first diagnosis; five years later, 10% of individuals with schizophrenia were employed. From systematic review - the most frequently reported disease characteristics that were negatively associated with employment were negative symptoms and cognitive symptoms. | (Bouwmans et al., 2015; Hakulinen et al., 2019; Holm et al., 2021) |
| Schizophrenia | Unstable housing | + | Cross-sectional study \| Expert opinion | Of the total participants (n = 1825, 63% with schizophrenia/schizoaffective disorder), one half were living in public or private rented housing (48.6%); 22.7% were waiting for public housing; 27.4% had changed housing in the previous year. Despite being the preferred form of housing, only 13.1% were living in their own home. One in 20 participants (5.2%) was currently homeless - 10 times the general population estimate of 0.5%; 12.8% had been homeless in the previous 12 months. Homelessness was enduring: those who had been homeless had spent considerable time so, with a mean of 155 days and a median of 99 days of homelessness over the past year. Because of the nature and impacts of their illness, people with severe and persistent mental illness often have a number of impairments which limit their ability to perform the tasks required to find and maintain stable and secure housing; for people with schizophrenia, these include auditory hallucinations, cognitive decline and impacts on motivation. | (Harvey et al., 2012; Morgan et al., 2012; Siskind, 2022) |
| Schizophrenia | Diet quality | - | Systematic review \| Narrative review \| Expert opinion | People with schizophrenia tend to have poor dietary patterns, including low intakes of fibre, fruit and vegetables; high intakes of sodium, sugar-sweetened beverages and convenience foods; as well as higher total energy intake compared to controls. Negative symptoms, such as apathy, lack of energy and motivation, prevent many people with schizophrenia spectrum disorders from activities such as grocery shopping and preparing a home-cooked meal; cognitive deficits also make the tasks required to prepare healthy meals more difficult. | (Dipasquale et al., 2013; Siskind, 2022; Teasdale et al., 2019; van Zonneveld et al., 2022) |
| Schizophrenia | Social support | - | Narrative review \| Cross-sectional study | Asociality, that is a reduction in the frequency of social interaction and the desire to form close relationships, is a negative symptom of schizophrenia. From cross-sectional study (n = 1825, 63% with schizophrenia/schizoaffective disorder), a large proportion of participants (69.3%) said that their illness made it hard to maintain close relationships. One-quarter (22.4%) felt socially isolated and lonely and a further 24.2% said that, despite having some friends, they were lonely for company. While most (86.5%) had one or more friends, 47.5% said they needed and would like to have more. Almost one-third (31.0%) lived alone and 40.6% of these would prefer to be living with someone else. Moreover, 13.3% of participants had no friends at all, 14.1% had no one to rely on and 15.4% had never had a confiding relationship. | (Morgan et al., 2012; Strauss & Cohen, 2017) |
| Schizophrenia | Antipsychotics | + | Systematic review | Standard management of schizophrenia includes the use of antipsychotic medications to help control acute psychotic episodes and prevent relapses, whereas maintenance therapy is used in the long term after patients have been stabilized. Two main classes of drugs—first- and second-generation antipsychotics (FGA and SGA)—are used to treat schizophrenia. | (Correll et al., 2022) |
| Screening | Physical diseases | - | Established | Screening for risk factors and diseases enables early intervention which can prevent diseases from developing |  |
| Screening | Diagnosis | + | Qualitative study | Mental health care professionals employed in the outpatient setting explained how they used screening tools in assessing symptoms and monitoring physical health among their clients. They explained how the thorough questioning about experienced physical symptoms helped them detect physical conditions, which they would otherwise have overlooked. | (Lerbaek et al., 2019) |
| Smoking | Physical diseases | + | Meta-analysis | Contributes primarily to cardiovascular disease, cancers and chronic respiratory diseases | (GBD 2019 Risk Factors Collaborators, 2020) for data visualisations, visit <https://vizhub.healthdata.org/gbd-compare> |
| Smoking | Alcohol use | + | Cross-sectional study | Tobacco smoking associated with alcohol dependence in study of 474 patients with schizophrenia; explained at least by cross reinforcement, alcohol and nicotine potentiating each other’s rewarding effects. Future studies detailing the reciprocal impact of tobacco and alcohol in schizophrenia would be of interest. | (Mallet et al., 2019) |
| Smoking | Income | - | Cross-sectional study | Study looking at 402 people with psychosis (180 with schizophrenia/schizoaffective disorder); smokers were more likely to go without basic necessities, including meals, illustrating the financial burden of supporting nicotine addiction. Smokers were also more likely to live with friends or be co-tenants; the financial consequences of smoking may make living alone unaffordable. | (Hahn et al., 2013) |
| Social support | Physical activity | + | Meta-analysis | Most frequently experienced socio-ecological barrier to exercise in people with schizophrenia was lack of support. | (Firth et al., 2016) |
| Social support | Antipsychotics | + | Cohort study | In a study of 547 patients with first-episode schizophrenia spectrum disorders, patients who did not receive any support from their key relative had significantly poorer adherence to medication, both at 1- and 2-year follow up. In a study of 112 patients with schizophrenia or schizoaffective disorder, compared to non/partial adherent participants, adherent participants showed greater perceived family involvement in pharmacological treatment. | (Baloush-Kleinman et al., 2011; Quach et al., 2009) |
| Social support | Treatment | + | Cross-sectional study | Perceived social support was associated with greater adherence to cardiovascular disease medication; for each 1% increase in social support, there was a 4.2% increase in medication adherence (odds ratio = 1.042, 95% confidence interval 1.015–1.070). | (Burton et al., 2020) |
| Social support | Primary care | + | Qualitative study | The relatives [of patients] perceived themselves as spokespersons who facilitated patients’ access to health care by accompanying them to appointments, asking questions and helping them with information about treatments, etc. | (Bjork Bramberg et al., 2018) |
| Social support | Stigma | - | Cross-sectional study | Among the 271 patients interviewed (89 with schizophrenia spectrum disorders), the number of relatives and friends, and perceived social support from relatives were significantly associated with internalized mental illness stigma: the larger the number of relatives and friends and the greater the support, the lower the stigma. | (Cullen et al., 2017) |
| Social support | Diet quality | + | Systematic review | Several studies highlighted how participants were supported (by family members, home-care staff, community centres) to maintain routines and provide structure, especially in terms of eating habits when mentally unwell. | (Balogun-Katung et al., 2021) |
| Stigma | Quality of care | - | Qualitative study | A perceived judgmental attitude, lack of respect, and absence of listening to patients were thought to impede rapport and trust between (primary care) providers and patients with a severe mental illness (SMI). (Primary care) clinicians believed patients were not interested in preventative care, had low expectations of patients following recommendations, self-described fatalistic prejudice - "this patient is going to die at an earlier age just from social unrest or living on the streets or the drugs they’re abusing" [so preventative care won't make much difference]. General medical clinicians expected individuals with schizophrenia to have lower levels of treatment adherence and competence in managing their health, and were less likely to refer for specialists and treatment. Clinicians described patients with SMI as challenging, mainly in regards to self-management; patients reported feeling “not heard,” “dismissed” and “not [taken] seriously”. Accounts of mental health care professionals sometimes choosing not to take action on physical health issues due to the severity and persistence of the mental problems among clients might reflect a latent discriminating attitude towards people with SMI embedded in the local cultures; participants to some extent confirmed this in their sporadic reflections about their contribution to continued stigma against people with SMI, even though this was depicted as unintentional. | (Kaufman et al., 2012; Knyahnytska et al., 2018; Lerbaek et al., 2019; Stone et al., 2019; Stumbo et al., 2018) |
| Stigma | Diagnostic overshadowing | + | Systematic review \| Cross-sectional study | Health professionals acknowledged that negative perceptions about mental health consumers remain pervasive in general healthcare settings; mental health consumers felt their physical complaints were minimised. Majority of participants [of survey on stigma and discrimination on people living with complex mental health issues] agreed that they had been unfairly denied help for their physical health problems, or had received inadequate or inappropriate healthcare, because of stigma about their mental health issues. | (Groot et al., 2020; Molloy et al., 2021) |
| Stigma | Unemployment | + | Cross-sectional study \| Narrative review | Negative discrimination was experienced by 209 (29%) of 724 participants in finding a job and 215 (29%) of 730 participants in keeping a job. Anticipated discrimination affected 469 (64%) participants in applying for work, training, or education. People with schizophrenia commonly report that stigma is one of the biggest barriers to them finding and keeping work. | (Marwaha & Johnson, 2004; Thornicroft et al., 2009) |
| Stigma | Unstable housing | + | Longitudinal study \| Narrative review | People with severe mental disorders who experienced less discrimination and stigma had better housing stability. Due to stigmatising attitudes, evidence of psychiatric disability can compromise housing applications. | (Browne & Courtney, 2007; Mejia-Lancheros et al., 2021) |
| Stigma | Social support | - | Cross-sectional study | Negative discrimination was experienced by 344 (47%) of 729 participants in making or keeping friends. Anticipated discrimination affected 402 (55%) participants in looking for a close relationship. | (Thornicroft et al., 2009) |
| Treatment | Premature mortality | - | Cohort study | In a cohort study of 105,018 patients with myocardial infarction, including 684 patients with schizophrenia, analyses of the associations of different cardiac therapy strategies with mortality rates revealed that patients with schizophrenia who were treated with any combination of triple therapy had mortality rates similar to those observed in the general population (hazard ratio = 1.05; 95% confidence interval, 0.43-2.52). In a study of people with first cancer diagnoses in 1990-2013, controlling for cancer treatment decreased the cancer-specific mortality risk in people with psychosis. | (Kugathasan et al., 2018; Manderbacka et al., 2017) |
| Treatment | Acute illness | - | Established | Receiving proper treatment prevents can prevent a disease from progressing to severe stages and the development of complications |  |
| Treatment | Primary care | + | Hypothetical | If effective treatment is provided, patients are likely to return to primary care providers for ongoing check ups and if new problems arise |  |
| Unemployment | Income | - | Established | Employment provides stable income; people who are unenployed often rely on government payments which equates to a low income |  |
| Unemployment | Social support | - | Cross-sectional study | Subjects following an occupation have a larger social network and report more social support by others. They do so because in particular colleagues at the workplace are perceived as an important source of emotional support. | (Ruesch et al., 2004) |
| Unstable housing | Diet quality | - | Qualitative study | Quote from client of supportive housing agency: "every meal you got on the street…was processed food…[we are] used to eating stuff that we didn’t cook ourself, we had to eat what was in front of us”. | (Stefancic et al., 2021) |
| Unstable housing | Physical activity | - | Qualitative study | The stress of having “their bodies in survival mode nonstop” and doing “a lot of walking” while homeless also led to a shift towards more sedentary behavior when in housing and made it difficult to engage in health-promoting behaviors; quote from peer specialist - "because they’ve been so beat up out there, once they get into housing, they just want to relax and don’t want to do much”. | (Stefancic et al., 2021) |
| Unstable housing | Stigma | + | Longitudinal study | People with severe mental disorders who who had less housing stability experienced worse stigma; authors discuss how experiences of homelessness contribute to stigma. | (Mejia-Lancheros et al., 2021) |
| Unstable housing | Primary care | - | Qualitative study | Both clients and service providers perceived that it was challenging to find and maintain a family physician in the context of unstable housing; not having a fixed address or phone number. | (Ross et al., 2015) |
| Unstable housing | Social support | - | Narrative review | If people with schizophrenia cannot maintain quality housing, they find it difficult to maintain supportive social relationships. | (Browne & Courtney, 2007) |

**Table S3: Variables in each feedback loop, with corresponding domain and moderators if applicable**

| **Feedback loops** | **Domain** | **Moderators** |
| --- | --- | --- |
| R1: Stigma, unstable housing | Social |  |
| R2: Smoking, alcohol use | Individual |  |
| R3: Physical diseases, physical activity | Individual |  |
| R4: Stigma, social support | Social |  |
| R5: Stigma, unstable housing, social support | Social |  |
| R6: Antipsychotics, smoking, alcohol use | Cross-domain |  |
| R7: Stigma, unemployment, social support | Social |  |
| R8: Stigma, unemployment, income, unstable housing | Social | Social support |
| R9: Treatment, primary care, screening, diagnosis | Healthcare |  |
| R10: Physical diseases, unemployment, income, physical activity | Cross-domain | Unstable housing |
| R11: Physical diseases, unemployment, income, diet quality | Cross-domain | Unstable housing; metabolic abnormalities |
| R12: Social support, diet quality, physical diseases, unemployment | Cross-domain | Metabolic abnormalities |
| R13: Social support, physical activity, physical diseases, unemployment | Cross-domain |  |
| R14: Stigma, quality of care, treatment, acute illness | Cross-domain | Antipsychotics |
| R15: Social support, antipsychotics, smoking, income, unstable housing | Cross-domain | Stigma |
| R16: Social support, antipsychotics, smoking, physical diseases, unemployment | Cross-domain | Alcohol use |
| R17: Stigma, diagnostic overshadowing, diagnosis, treatment, acute illness | Cross-domain |  |
| R18: Physical diseases, unemployment, income, primary care, screening | Cross-domain |  |
| R19: Stigma, quality of care, screening, diagnosis, treatment, acute illness | Cross-domain |  |
| B1: Antipsychotics, metabolic abnormalities | Cross-domain | Diet quality |
| B2: Physical diseases, primary care, screening | Cross-domain |  |
| B3: Social support, antipsychotics, diet quality, physical diseases, unemployment | Cross-domain |  |
| B4: Social support, antipsychotics, metabolic abnormalities, physical diseases, unemployment | Cross-domain | Physical activity |

**References**

Ahn, S., Choi, Y., Choi, W., Jo, Y. T., Kim, H., Lee, J., & Joo, S. W. (2021). Effects of comorbid alcohol use disorder on the clinical outcomes of first-episode schizophrenia: a nationwide population-based study. *Ann Gen Psychiatry*, *20*(1), 32. <https://doi.org/10.1186/s12991-021-00353-3>

Ali, S., Santomauro, D., Ferrari, A. J., & Charlson, F. (2022). Excess mortality in severe mental disorders: A systematic review and meta-regression. *Journal of Psychiatric Research*, *149*, 97-105. <https://doi.org/https://doi.org/10.1016/j.jpsychires.2022.02.036>

Attar, R., Jensen, S. E., Nielsen, R. E., Polcwiartek, C., Andell, P., Pedersen, C. T., & Kragholm, K. (2020). Time Trends in the Use of Coronary Procedures, Guideline-Based Therapy, and All-Cause Mortality following the Acute Coronary Syndrome in Patients with Schizophrenia. *Cardiology*, *145*(7), 401-409. <https://doi.org/10.1159/000507044>

Balogun-Katung, A., Carswell, C., Brown, J. V. E., Coventry, P., Ajjan, R., Alderson, S., Bellass, S., Boehnke, J. R., Holt, R., Jacobs, R., Kellar, I., Kitchen, C., Lister, J., Peckham, E., Shiers, D., Siddiqi, N., Wright, J., Young, B., Taylor, J., & team, D. r. (2021). Exploring the facilitators, barriers, and strategies for self-management in adults living with severe mental illness, with and without long-term conditions: A qualitative evidence synthesis. *PloS One*, *16*(10), e0258937. <https://doi.org/10.1371/journal.pone.0258937>

Baloush-Kleinman, V., Levine, S. Z., Roe, D., Shnitt, D., Weizman, A., & Poyurovsky, M. (2011). Adherence to antipsychotic drug treatment in early-episode schizophrenia: a six-month naturalistic follow-up study. *Schizophrenia Research*, *130*(1-3), 176-181. <https://doi.org/10.1016/j.schres.2011.04.030>

Bjork Bramberg, E., Torgerson, J., Norman Kjellstrom, A., Welin, P., & Rusner, M. (2018). Access to primary and specialized somatic health care for persons with severe mental illness: a qualitative study of perceived barriers and facilitators in Swedish health care. *BMC Family Practice*, *19*(1), 12. <https://doi.org/10.1186/s12875-017-0687-0>

Bouwmans, C., de Sonneville, C., Mulder, C. L., & Hakkaart-van Roijen, L. (2015). Employment and the associated impact on quality of life in people diagnosed with schizophrenia. *Neuropsychiatric Disease and Treatment*, *11*, 2125-2142. <https://doi.org/10.2147/NDT.S83546>

Browne, G., & Courtney, M. (2007). Schizophrenia housing and supportive relationships. *International Journal of Mental Health Nursing*, *16*(2), 73-80. <https://doi.org/10.1111/j.1447-0349.2006.00447.x>

Burton, A., Walters, K., Marston, L., & Osborn, D. (2020). Is there an association between perceived social support and cardiovascular health behaviours in people with severe mental illnesses? *Social Psychiatry and Psychiatric Epidemiology*. <https://doi.org/10.1007/s00127-020-01879-9>

Chochinov, H. M., Martens, P. J., Prior, H. J., Fransoo, R., Burland, E., & Need To Know, T. (2009). Does a diagnosis of schizophrenia reduce rates of mammography screening? A Manitoba population-based study. *Schizophrenia Research*, *113*(1), 95-100. <https://doi.org/10.1016/j.schres.2009.04.022>

Correll, C. U., Detraux, J., De Lepeleire, J., & De Hert, M. (2015). Effects of antipsychotics, antidepressants and mood stabilizers on risk for physical diseases in people with schizophrenia, depression and bipolar disorder. *World Psychiatry*, *14*(2), 119-136. <https://doi.org/10.1002/wps.20204>

Correll, C. U., Martin, A., Patel, C., Benson, C., Goulding, R., Kern-Sliwa, J., Joshi, K., Schiller, E., & Kim, E. (2022). Systematic literature review of schizophrenia clinical practice guidelines on acute and maintenance management with antipsychotics. *Schizophrenia*, *8*(1), 5. <https://doi.org/10.1038/s41537-021-00192-x>

Crawford, M. J., Jayakumar, S., Lemmey, S. J., Zalewska, K., Patel, M. X., Cooper, S. J., & Shiers, D. (2014). Assessment and treatment of physical health problems among people with schizophrenia: National cross-sectional study. *The British Journal of Psychiatry*, *205*(6), 473-477. <https://doi.org/10.1192/bjp.bp.113.142521>

Cullen, B. A. M., Mojtabai, R., Bordbar, E., Everett, A., Nugent, K. L., & Eaton, W. W. (2017). Social network, recovery attitudes and internal stigma among those with serious mental illness. *International Journal of Social Psychiatry*, *63*(5), 448-458. <https://doi.org/10.1177/0020764017712302>

de Leon, J., & Diaz, F. J. (2005). A meta-analysis of worldwide studies demonstrates an association between schizophrenia and tobacco smoking behaviors. *Schizophrenia Research*, *76*(2-3), 135-157. <https://doi.org/10.1016/j.schres.2005.02.010>

Dickerson, F., Schroeder, J., Katsafanas, E., Khushalani, S., Origoni, A. E., Savage, C., Schweinfurth, L., Stallings, C. R., Sweeney, K., & Yolken, R. H. (2018). Cigarette Smoking by Patients With Serious Mental Illness, 1999-2016: An Increasing Disparity. *Psychiatric Services*, *69*(2), 147-153. <https://doi.org/10.1176/appi.ps.201700118>

Dipasquale, S., Pariante, C. M., Dazzan, P., Aguglia, E., McGuire, P., & Mondelli, V. (2013). The dietary pattern of patients with schizophrenia: A systematic review. *Journal of Psychiatric Research*, *47*(2), 197-207. <https://doi.org/10.1016/j.jpsychires.2012.10.005>

El-Mallakh, P. (2007). Doing my best: poverty and self-care among individuals with schizophrenia and diabetes mellitus. *Archives of Psychiatric Nursing*, *21*(1), 49-60; discussion 61-43. <https://doi.org/10.1016/j.apnu.2006.10.004>

El Abdellati, K., De Picker, L., & Morrens, M. (2020). Antipsychotic Treatment Failure: A Systematic Review on Risk Factors and Interventions for Treatment Adherence in Psychosis. *Frontiers in Neuroscience*, *14*, 531763. <https://doi.org/10.3389/fnins.2020.531763>

Firth, J., Rosenbaum, S., Stubbs, B., Gorczynski, P., Yung, A. R., & Vancampfort, D. (2016). Motivating factors and barriers towards exercise in severe mental illness: a systematic review and meta-analysis. *Psychological Medicine*, *46*(14), 2869-2881. <https://doi.org/10.1017/S0033291716001732>

GBD 2019 Mental Disorders Collaborators. (2022). Global, regional, and national burden of 12 mental disorders in 204 countries and territories, 1990–2019: a systematic analysis for the Global Burden of Disease Study 2019. *The Lancet Psychiatry*, *9*(2), 137-150. <https://doi.org/10.1016/s2215-0366(21)00395-3>

GBD 2019 Risk Factors Collaborators. (2020). Global burden of 87 risk factors in 204 countries and territories, 1990–2019: a systematic analysis for the Global Burden of Disease Study 2019. *The Lancet*, *396*(10258), 1223-1249. <https://doi.org/10.1016/s0140-6736(20)30752-2>

Gerlinger, G., Hauser, M., De Hert, M., Lacluyse, K., Wampers, M., & Correll, C. U. (2013). Personal stigma in schizophrenia spectrum disorders: a systematic review of prevalence rates, correlates, impact and interventions. *World psychiatry : official journal of the World Psychiatric Association (WPA)*, *12*(2), 155-164. <https://doi.org/10.1002/wps.20040>

Groot, C., Rehm, I., Andrews, C., Hobern, B., Morgan, R., Green, H., Sweeney, L., & Blanchard, M. (2020). *Report on Findings from the Our Turn to Speak Survey: Understanding the impact of stigma and discrimination on people living with complex mental health issues*.

Hahn, L., Rigby, A., & Galletly, C. (2013). Determinants of high rates of smoking among people with psychosis living in a socially disadvantaged region in South Australia. *Australian and New Zealand Journal of Psychiatry*, *48*(1), 70-79. <https://doi.org/10.1177/0004867413491158>

Hakulinen, C., McGrath, J. J., Timmerman, A., Skipper, N., Mortensen, P. B., Pedersen, C. B., & Agerbo, E. (2019). The association between early-onset schizophrenia with employment, income, education, and cohabitation status: nationwide study with 35 years of follow-up. *Social Psychiatry and Psychiatric Epidemiology*, *54*(11), 1343-1351. <https://doi.org/10.1007/s00127-019-01756-0>

Happell, B., Ewart, S. B., Bocking, J., Platania-Phung, C., & Stanton, R. (2016). 'That red flag on your file': misinterpreting physical symptoms as mental illness. *Journal of Clinical Nursing*, *25*(19-20), 2933-2942. <https://doi.org/10.1111/jocn.13355>

Harvey, C., Killackey, E., Groves, A., & Herrman, H. (2012). A place to live: Housing needs for people with psychotic disorders identified in the second Australian national survey of psychosis. *Australian and New Zealand Journal of Psychiatry*, *46*(9), 840-850. <https://doi.org/10.1177/0004867412449301>

Holm, M., Taipale, H., Tanskanen, A., Tiihonen, J., & Mitterdorfer-Rutz, E. (2021). Employment among people with schizophrenia or bipolar disorder: A population-based study using nationwide registers. *Acta Psychiatrica Scandinavica*, *143*(1), 61-71. <https://doi.org/10.1111/acps.13254>

Hunt, G. E., Large, M. M., Cleary, M., Lai, H. M. X., & Saunders, J. B. (2018). Prevalence of comorbid substance use in schizophrenia spectrum disorders in community and clinical settings, 1990-2017: Systematic review and meta-analysis. *Drug and Alcohol Dependence*, *191*, 234-258. <https://doi.org/10.1016/j.drugalcdep.2018.07.011>

Jauhar, S., Johnstone, M., & McKenna, P. J. (2022). Schizophrenia. *The Lancet*, *399*(10323), 473-486. <https://doi.org/10.1016/s0140-6736(21)01730-x>

Jonsdottir, H., Opjordsmoen, S., Birkenaes, A. B., Simonsen, C., Engh, J. A., Ringen, P. A., Vaskinn, A., Friis, S., Sundet, K., & Andreassen, O. A. (2013). Predictors of medication adherence in patients with schizophrenia and bipolar disorder. *Acta Psychiatrica Scandinavica*, *127*(1), 23-33. <https://doi.org/10.1111/j.1600-0447.2012.01911.x>

Kaufman, E. A., McDonell, M. G., Cristofalo, M. A., & Ries, R. K. (2012). Exploring barriers to primary care for patients with severe mental illness: frontline patient and provider accounts. *Issues in Mental Health Nursing*, *33*(3), 172-180. <https://doi.org/10.3109/01612840.2011.638415>

Khokhar, J. Y., Dwiel, L. L., Henricks, A. M., Doucette, W. T., & Green, A. I. (2018). The link between schizophrenia and substance use disorder: A unifying hypothesis. *Schizophrenia Research*, *194*, 78-85. <https://doi.org/10.1016/j.schres.2017.04.016>

Knyahnytska, Y., Williams, C., Dale, C., & Webster, F. (2018). Changing the Conversation: Diabetes Management in Adults With Severe Mental Illnesses and Type 2 Diabetes. *Can J Diabetes*, *42*(6), 595-602. <https://doi.org/10.1016/j.jcjd.2018.02.001>

Kontopantelis, E., Olier, I., Planner, C., Reeves, D., Ashcroft, D. M., Gask, L., Doran, T., & Reilly, S. (2015). Primary care consultation rates among people with and without severe mental illness: a UK cohort study using the Clinical Practice Research Datalink. *BMJ Open*, *5*(12), e008650. <https://doi.org/10.1136/bmjopen-2015-008650>

Koskinen, J., Lohonen, J., Koponen, H., Isohanni, M., & Miettunen, J. (2009). Prevalence of alcohol use disorders in schizophrenia--a systematic review and meta-analysis. *Acta Psychiatrica Scandinavica*, *120*(2), 85-96. <https://doi.org/10.1111/j.1600-0447.2009.01385.x>

Kugathasan, P., Horsdal, H. T., Aagaard, J., Jensen, S. E., Laursen, T. M., & Nielsen, R. E. (2018). Association of secondary preventive cardiovascular treatment after myocardial infarction with mortality among patients with schizophrenia. *JAMA Psychiatry*, *75*(12), 1234-1240. <https://doi.org/10.1001/jamapsychiatry.2018.2742>

Lerbaek, B., Jorgensen, R., Aagaard, J., Nordgaard, J., & Buus, N. (2019). Mental health care professionals' accounts of actions and responsibilities related to managing physical health among people with severe mental illness. *33*(2), 174-181. <https://doi.org/10.1016/j.apnu.2018.11.006>

MacEwan, J. P., Forma, F. M., Shafrin, J., Hatch, A., Lakdawalla, D. N., & Lindenmayer, J.-P. (2016). Patterns of Adherence to Oral Atypical Antipsychotics Among Patients Diagnosed with Schizophrenia. *Journal of Managed Care & Specialty Pharmacy*, *22*(11), 1349-1361. <https://doi.org/10.18553/jmcp.2016.22.11.1349>

Mallet, J., Le Strat, Y., Schurhoff, F., Mazer, N., Portalier, C., Andrianarisoa, M., Aouizerate, B., Berna, F., Brunel, L., Capdevielle, D., Chereau, I., D'Amato, T., Dubreucq, J., Faget, C., Gabayet, F., Honciuc, R. M., Lancon, C., Llorca, P. M., Misdrahi, D., Rey, R., Roux, P., Schandrin, A., Urbach, M., Vidailhet, P., Fond, G., & Dubertret, C. (2019). Tobacco smoking is associated with antipsychotic medication, physical aggressiveness, and alcohol use disorder in schizophrenia: results from the FACE-SZ national cohort. *269*(4), 449-457. <https://doi.org/10.1007/s00406-018-0873-7>

Manderbacka, K., Arffman, M., Suvisaari, J., Ahlgren-Rimpilainen, A., Lumme, S., Keskimaki, I., & Pukkala, E. (2017). Effect of stage, comorbidities and treatment on survival among cancer patients with or without mental illness. *British Journal of Psychiatry*, *211*(5), 304-309. <https://doi.org/10.1192/bjp.bp.117.198952>

Martens, P. J., Chochinov, H. M., Prior, H. J., Fransoo, R., Burland, E., & Need To Know, T. (2009). Are cervical cancer screening rates different for women with schizophrenia? A Manitoba population-based study. *Schizophrenia Research*, *113*(1), 101-106. <https://doi.org/10.1016/j.schres.2009.04.015>

Marwaha, S., & Johnson, S. (2004). Schizophrenia and employment - a review. *Social Psychiatry and Psychiatric Epidemiology*, *39*(5), 337-349. <https://doi.org/10.1007/s00127-004-0762-4>

McDevitt, J., Snyder, M., Miller, A., & Wilbur, J. (2006). Perceptions of Barriers and Benefits to Physical Activity Among Outpatients in Psychiatric Rehabilitation [<https://doi.org/10.1111/j.1547-5069.2006.00077.x>]. *Journal of Nursing Scholarship*, *38*(1), 50-55. <https://doi.org/https://doi.org/10.1111/j.1547-5069.2006.00077.x>

Mejia-Lancheros, C., Lachaud, J., Woodhall-Melnik, J., O'Campo, P., Hwang, S. W., & Stergiopoulos, V. (2021). Longitudinal interrelationships of mental health discrimination and stigma with housing and well-being outcomes in adults with mental illness and recent experience of homelessness. *Social Science and Medicine*, *268*, 113463. <https://doi.org/10.1016/j.socscimed.2020.113463>

Molloy, R., Brand, G., Munro, I., & Pope, N. (2021). Seeing the complete picture: A systematic review of mental health consumer and health professional experiences of diagnostic overshadowing. *Journal of Clinical Nursing*. <https://doi.org/10.1111/jocn.16151>

Morgan, V. A., Waterreus, A., Carr, V., Castle, D., Cohen, M., Harvey, C., Galletly, C., Mackinnon, A., McGorry, P., McGrath, J. J., Neil, A. L., Saw, S., Badcock, J. C., Foley, D. L., Waghorn, G., Coker, S., & Jablensky, A. (2016). Responding to challenges for people with psychotic illness: Updated evidence from the Survey of High Impact Psychosis. *Australian and New Zealand Journal of Psychiatry*, *51*(2), 124-140. <https://doi.org/10.1177/0004867416679738>

Morgan, V. A., Waterreus, A., Jablensky, A., Mackinnon, A., McGrath, J. J., Carr, V., Bush, R., Castle, D., Cohen, M., Harvey, C., Galletly, C., Stain, H. J., Neil, A. L., McGorry, P., Hocking, B., Shah, S., & Saw, S. (2012). People living with psychotic illness in 2010: The second Australian national survey of psychosis. *Australian and New Zealand Journal of Psychiatry*, *46*(8), 735-752. <https://doi.org/10.1177/0004867412449877>

Murphy, K. A., Daumit, G. L., McGinty, E. E., Stone, E. M., & Kennedy-Hendricks, A. (2021). Predictors of cancer screening among Black and White Maryland Medicaid enrollees with serious mental illness. *Psycho-Oncology*. <https://doi.org/10.1002/pon.5815>

Pillinger, T., McCutcheon, R. A., Vano, L., Mizuno, Y., Arumuham, A., Hindley, G., Beck, K., Natesan, S., Efthimiou, O., Cipriani, A., & Howes, O. D. (2020). Comparative effects of 18 antipsychotics on metabolic function in patients with schizophrenia, predictors of metabolic dysregulation, and association with psychopathology: a systematic review and network meta-analysis. *The Lancet Psychiatry*, *7*(1), 64-77. <https://doi.org/10.1016/s2215-0366(19)30416-x>

Quach, P. L., Mors, O., Christensen, T. O., Krarup, G., Jorgensen, P., Bertelsen, M., Jeppesen, P., Petersen, L., Thorup, A., & Nordentoft, M. (2009). Predictors of poor adherence to medication among patients with first-episode schizophrenia-spectrum disorder. *Early Interv Psychiatry*, *3*(1), 66-74. <https://doi.org/10.1111/j.1751-7893.2008.00108.x>

Ronaldson, A., Elton, L., Jayakumar, S., Jieman, A., Halvorsrud, K., & Bhui, K. (2020). Severe mental illness and health service utilisation for nonpsychiatric medical disorders: A systematic review and meta-analysis. *PLoS Medicine*, *17*(9), e1003284. <https://doi.org/10.1371/journal.pmed.1003284>

Ross, L. E., Vigod, S., Wishart, J., Waese, M., Spence, J. D., Oliver, J., Chambers, J., Anderson, S., & Shields, R. (2015). Barriers and facilitators to primary care for people with mental health and/or substance use issues: a qualitative study. *BMC Family Practice*, *16*, 135. <https://doi.org/10.1186/s12875-015-0353-3>

Ruesch, P., Graf, J., Meyer, P. C., Rossler, W., & Hell, D. (2004). Occupation, social support and quality of life in persons with schizophrenic or affective disorders. *Social Psychiatry and Psychiatric Epidemiology*, *39*(9), 686-694. <https://doi.org/10.1007/s00127-004-0812-y>

Schulman-Marcus, J., Goyal, P., Swaminathan, R. V., Feldman, D. N., Wong, S.-C., Singh, H. S., Minutello, R. M., Bergman, G., & Kim, L. K. (2016). Comparison of Trends in Incidence, Revascularization, and In-Hospital Mortality in ST-Elevation Myocardial Infarction in Patients With Versus Without Severe Mental Illness. *The American Journal of Cardiology*, *117*(9), 1405-1410. <https://doi.org/https://doi.org/10.1016/j.amjcard.2016.02.006>

Schulze, B., & Angermeyer, M. C. (2003). Subjective experiences of stigma. A focus group study of schizophrenic patients, their relatives and mental health professionals. *Social Science and Medicine*, *56*(2), 299-312. <https://doi.org/https://doi.org/10.1016/S0277-9536(02)00028-X>

Singh, R., Bansal, Y., Medhi, B., & Kuhad, A. (2019). Antipsychotics-induced metabolic alterations: Recounting the mechanistic insights, therapeutic targets and pharmacological alternatives. *European Journal of Pharmacology*, *844*, 231-240. <https://doi.org/10.1016/j.ejphar.2018.12.003>

Siskind, D. (2022). *Submission to Inquiry into the opportunities to improve mental health outcomes for Queenslanders*. <https://www.parliament.qld.gov.au/Work-of-Committees/Committees/Committee-Details?cid=226&id=4143>

Solmi, M., Fiedorowicz, J., Poddighe, L., Delogu, M., Miola, A., Høye, A., Heiberg, I. H., Stubbs, B., Smith, L., Larsson, H., Attar, R., Nielsen, R. E., Cortese, S., Shin, J. I., Fusar-Poli, P., Firth, J., Yatham, L. N., Carvalho, A. F., Castle, D. J., Seeman, M. V., & Correll, C. U. (2021). Disparities in Screening and Treatment of Cardiovascular Diseases in Patients With Mental Disorders Across the World: Systematic Review and Meta-Analysis of 47 Observational Studies. *American Journal of Psychiatry*, appi.ajp.2021.21010031. <https://doi.org/10.1176/appi.ajp.2021.21010031>

Solmi, M., Murru, A., Pacchiarotti, I., Undurraga, J., Veronese, N., Fornaro, M., Stubbs, B., Monaco, F., Vieta, E., Seeman, M. V., Correll, C. U., & Carvalho, A. F. (2017). Safety, tolerability, and risks associated with first- and second-generation antipsychotics: a state-of-the-art clinical review. *Therapeutics and Clinical Risk Management*, *13*, 757-777. <https://doi.org/10.2147/TCRM.S117321>

Solmi, M., Tiihonen, J., Lahteenvuo, M., Tanskanen, A., Correll, C. U., & Taipale, H. (2021). Antipsychotics Use Is Associated With Greater Adherence to Cardiometabolic Medications in Patients With Schizophrenia: Results From a Nationwide, Within-subject Design Study. *Schizophrenia Bulletin*. <https://doi.org/10.1093/schbul/sbab087>

Stefancic, A., Bochicchio, L., Svehaug, K., Alvi, T., & Cabassa, L. J. (2021). “We Die 25 Years Sooner:” Addressing Physical Health Among Persons with Serious Mental Illness in Supportive Housing. *Community Mental Health Journal*. <https://doi.org/10.1007/s10597-020-00752-y>

Stone, E. M., Chen, L. N., Daumit, G. L., Linden, S., & McGinty, E. E. (2019). General Medical Clinicians' Attitudes Toward People with Serious Mental Illness: A Scoping Review. <https://doi.org/10.1007/s11414-019-09652-w>

Strauss, G. P., & Cohen, A. S. (2017). A Transdiagnostic Review of Negative Symptom Phenomenology and Etiology. *Schizophrenia Bulletin*, *43*(4), 712-719. <https://doi.org/10.1093/schbul/sbx066>

Stubbs, B., Firth, J., Berry, A., Schuch, F. B., Rosenbaum, S., Gaughran, F., Veronesse, N., Williams, J., Craig, T., Yung, A. R., & Vancampfort, D. (2016). How much physical activity do people with schizophrenia engage in? A systematic review, comparative meta-analysis and meta-regression. *Schizophrenia Research*, *176*(2-3), 431-440. <https://doi.org/10.1016/j.schres.2016.05.017>

Stumbo, S. P., Yarborough, B. J. H., Yarborough, M. T., & Green, C. A. (2018). Perspectives on Providing And Receiving Preventive Health Care From Primary Care Providers and Their Patients With Mental Illnesses. *American Journal of Health Promotion*, *32*(8), 1730-1739. <https://doi.org/10.1177/0890117118763233>

Teasdale, S. B., Ward, P. B., Samaras, K., Firth, J., Stubbs, B., Tripodi, E., & Burrows, T. L. (2019). Dietary intake of people with severe mental illness: systematic review and meta-analysis. *British Journal of Psychiatry*, *214*(5), 251-259. <https://doi.org/10.1192/bjp.2019.20>

Tham, M. S. P., Jones, S. G., Chamberlain, J. A., & Castle, D. J. (2009). The impact of psychotropic weight gain on people with psychosis – patient perspectives and attitudes. *Journal of Mental Health*, *16*(6), 771-779. <https://doi.org/10.1080/09638230701496352>

Thornicroft, G., Brohan, E., Rose, D., Sartorius, N., & Leese, M. (2009). Global pattern of experienced and anticipated discrimination against people with schizophrenia: a cross-sectional survey. *The Lancet*, *373*(9661), 408-415. <https://doi.org/10.1016/s0140-6736(08)61817-6>

van Zonneveld, S. M., Haarman, B. C. M., van den Oever, E. J., Nuninga, J. O., & Sommer, I. E. C. (2022). Unhealthy diet in schizophrenia spectrum disorders. *Curr Opin Psychiatry*, *35*(3), 177-185. <https://doi.org/10.1097/YCO.0000000000000791>

Vancampfort, D., De Hert, M., Skjerven, L. H., Gyllensten, A. L., Parker, A., Mulders, N., Nyboe, L., Spencer, F., & Probst, M. (2012). International Organization of Physical Therapy in Mental Health consensus on physical activity within multidisciplinary rehabilitation programmes for minimising cardio-metabolic risk in patients with schizophrenia. *Disability and Rehabilitation*, *34*(1), 1-12. <https://doi.org/10.3109/09638288.2011.587090>

Vancampfort, D., Knapen, J., Probst, M., Scheewe, T., Remans, S., & De Hert, M. (2012). A systematic review of correlates of physical activity in patients with schizophrenia. *Acta Psychiatrica Scandinavica*, *125*(5), 352-362. <https://doi.org/10.1111/j.1600-0447.2011.01814.x>

Vancampfort, D., Sweers, K., Probst, M., Maurissen, K., Knapen, J., Minguet, P., & De Hert, M. (2011). Association of the metabolic syndrome with physical activity performance in patients with schizophrenia. *Diabetes and Metabolism*, *37*(4), 318-323. <https://doi.org/10.1016/j.diabet.2010.12.007>

Waghorn, G., Lloyd, C., Abraham, B., Silvester, D., & Chant, D. (2008). Comorbid physical health conditions hinder employment among people with psychiatric disabilities. *Psychiatr Rehabil J*, *31*(3), 243-246. <https://doi.org/10.2975/31.3.2008.243.246>

Ward, H. B., Beermann, A., Nawaz, U., Halko, M. A., Janes, A. C., Moran, L. V., & Brady, R. O., Jr. (2022). Evidence for Schizophrenia-Specific Pathophysiology of Nicotine Dependence. *Front Psychiatry*, *13*, 804055. <https://doi.org/10.3389/fpsyt.2022.804055>

Weiden, P. J., Mackell, J. A., & McDonnell, D. D. (2004). Obesity as a risk factor for antipsychotic noncompliance. *Schizophrenia Research*, *66*(1), 51-57. <https://doi.org/10.1016/s0920-9964(02)00498-x>

Wu, B. J., Chen, H. K., & Lee, S. M. (2013). Do atypical antipsychotics really enhance smoking reduction more than typical ones?: the effects of antipsychotics on smoking reduction in patients with schizophrenia. *Journal of Clinical Psychopharmacology*, *33*(3), 319-328. <https://doi.org/10.1097/JCP.0b013e31828b2575>

Wu, B. J., & Lan, T. H. (2017). Predictors of smoking reduction outcomes in a sample of 287 patients with schizophrenia spectrum disorders. *European Archives of Psychiatry and Clinical Neuroscience*, *267*(1), 63-72. <https://doi.org/10.1007/s00406-015-0636-7>
